# Supplementary material for: Determinants of sensitivity to HER2-targeted antibody drug conjugates in urothelial cancer
Source: Nat Commun. 2025 Dec 20;17:919. doi: 10.1038/s41467-025-67643-2 (PMC12830848; doi:10.1038/s41467-025-67643-2)

Determinants of Sensitivity to HER2-targeted Antibody Drug Conjugates in Urothelial Cancer

Ziyu Chen[1,2#], Xinran Tang[1,2#], Jordan E. Eichholz[3], Andrew Mcpherson[3,4], Jasmine Thomas[5], Karan Nagar[5], Naryan Rustgi[1], John R. Christin[6], Fengshen Kuo[1,5], Sizhi Gao[1], Hui Jiang[1,5], Jiaqian Luo[1,2], Irina Ostrovnaya[7], Merve Basar[8], Eugene Pietzak[9], Jonathan A. Coleman[9], Michael F. Berger[8,10], Elisa de Stanchina[11], Sohrab P. Shah[3,4], Neeman Mohibullah[12], David H. Aggen[13], Jonathan E. Rosenberg[13], Sarat Chandarlapaty[1], Michael M. Shen[6], Hikmat Al-Ahmadie[8], Gopa Iyer[13], Kwanghee Kim[5], David B. Solit[*1,10,13]

[1] Human Oncology and Pathogenesis Program, Memorial Sloan Kettering Cancer Center, New York, NY 10065, USA.
[2] Weill Cornell Medicine, Graduate School of Medical Sciences, New York, NY 10065, USA.
[3] Computational Oncology, Department of Epidemiology and Biostatistics, Memorial Sloan Kettering Cancer Center, New York, NY 10065, USA
[4] Halvorsen Center for Computational Oncology, Memorial Sloan Kettering Cancer Center, New York, NY 10065, USA.
[5] Department of Surgery, Memorial Sloan Kettering Cancer Center, New York, NY 10065, USA
[6] Department of Medicine, Genetics and Development, Urology, and Systems Biology, Herbert Irving Comprehensive Cancer Center, Columbia University Vagelos College of Physicians and Surgeons, New York, NY 10032, USA
[7] Biostatistics, Department of Epidemiology-Biostatistics, Memorial Sloan Kettering Cancer Center, New York, NY 10017, USA.
[8] Department of Pathology and Laboratory Medicine, Memorial Sloan Kettering Cancer Center, New York, NY 10065, USA.
[9] Urology Service, Department of Urology, Memorial Sloan Kettering Cancer Center, New York, NY 10065, USA.
[10] Marie-Josée and Henry R. Kravis Center for Molecular Oncology, Memorial Sloan Kettering Cancer Center, New York, NY 10065, USA.
[11] Antitumor Assessment Core Facility, Memorial Sloan Kettering Cancer Center, New York, NY 10065, USA
[12] Integrated Genomics Operation, Memorial Sloan Kettering Cancer Center, New York, NY 10065, USA
[13] Genitourinary Oncology Service, Department of Medicine, Memorial Sloan Kettering Cancer Center, New York, NY 10065, USA.

#Contributed equally
Key Words: HER2, bladder cancer, urothelial cancer, trastuzumab deruxtecan, neratinib

[*]Correspondence:
David B. Solit, MD
Memorial Sloan Kettering Cancer Center
1275 York Ave,
New York, NY 10065
646-888-2641
solitd@mskcc.org

Figure S1. *ERBB2* as a therapeutic target in bladder and upper tract urothelial cancer. A. Fraction of patients with *ERBB2* amplification (left) or mutation (right) as a function of cancer type in the MSK-IMPACT cohort (n = 42,515). **B.** Prevalence of *ERBB2* mutation/amplification in patients with a bladder (n = 1865) versus upper tract (n = 381) primary site. Bladder UC: bladder urothelial cancer, UTUC: upper tract urothelial cancer. **C.** Lollipop plot of *ERBB2* hotspot mutations in Bladder UC versus UTUC, with the S310F/Y mutation highlighted. **D.** Volcano plot of APOBEC-associated hotspot mutations in urothelial cancers, with the S310 mutation highlighted by the red arrow. The Y axis represents the $\log_2$[Odds ratio] and *P* value (from two-sided Fisher's exact test) comparing samples with and without dominant APOBEC signatures. Genes with adjusted p-value (q-value) <0.1 were defined as significant and colored red. The size of the circles represents the frequency of the mutation in the cohort. **E.** Percentages of tumors with *ERBB2* total copy number (TCN)>20 in urothelial carcinoma (UC), breast cancer (BRCA), endometrial cancer (UCEC), esophagogastric cancer (ESCA) and non-small cell lung cancer (NSCLC). Source data for A-D are provided in Source Data.

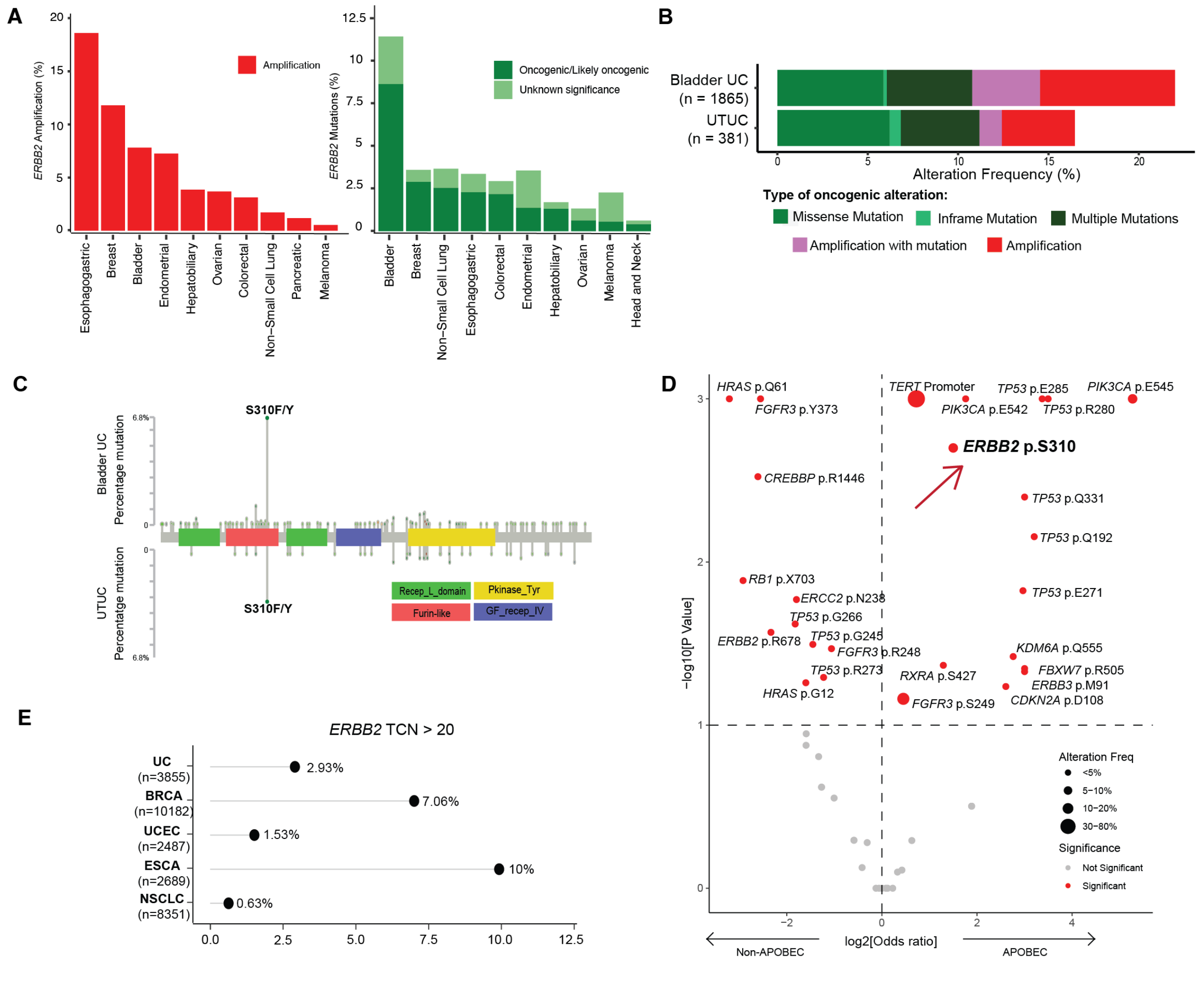

Figure S2. Landscape of *ERBB2* alterations in urothelial cancers stratified by clinical and pathologic features. A. Correlation of *ERBB2* alteration with patient demographic data including sex, race, and smoking status in patients with urothelial cancer. **B.** Frequency of oncogenic/likely oncogenic alterations in *ERBB2*, *FGFR3*, *PIK3CA* and *TSC1* stratified by disease state (low-grade primary tumors, non-invasive and invasive high-grade primary tumors, and metastatic sites). Significance is labeled as adjusted p value (q value): *q <0.05, **q <0.01, ***q <0.001. **C.** Frequency of oncogenic/likely oncogenic *ERBB2* alterations stratified by histologic subtype. NI-LGPUC: non-invasive, low grade papillary urothelial carcinoma, NI-HGPUC: non-invasive, high grade papillary urothelial carcinoma, CIS: carcinoma in situ, UC-NOS: urothelial carcinoma not otherwise specified. **D.** Phylogenic analysis of the primary and metastatic tumors from two representative patients with urothelial cancer with discordant *ERBB2* mutational status between primary and metastatic disease sites. The mutation matrix colored as trunk (dark green) or exclusive (light green) to the primary or metastatic site with cancer cell fraction denoted by shades of blue. Numbers indicate shared or private mutation counts. Source data for A-C are provided in Source Data.

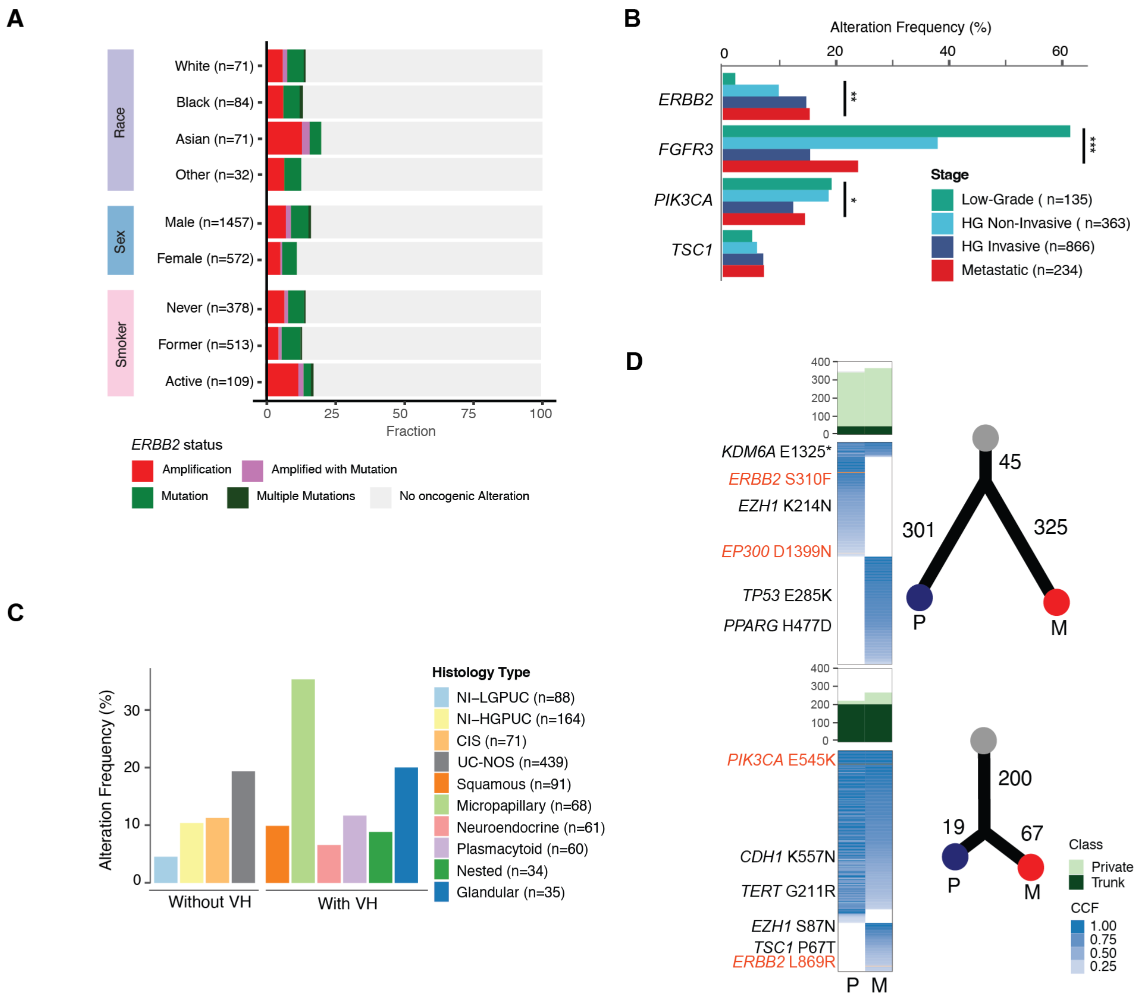

Figure S3. Genomic and clinical characterization of *ERBB2* mutated and amplified urothelial cancers. A. Volcano plots of co-altered genes in urothelial cancers with *ERBB2* amplification and/or mutation (left – both amplification and mutation, middle: amplification only, right: mutation only). The odds ratio and p value represent Chi-squared tests comparing samples with oncogenic/likely oncogenic *ERBB2* alteration vs samples without (denoted as *ERBB2* WT in the figure). Genes with adjusted p-value (q-value) <0.05 were defined as significant and colored red. The sizes of the circle represent the number of altered cases of that gene in the *ERBB2* altered groups. **B.** Tumor mutation burden (left) and fraction of genome altered (right) of urothelial cancers stratified by *ERBB2* status. Significance denoted as ** p<0.01, *** p<0.001, n.s. not significant**. C.** Comparison of *ERBB2* mRNA expression z-scores relative to all samples and HER2 protein level in urothelial cancer (UC, top) and BRCA (breast cancer, bottom) using data from the The Cancer Genome Atlas (TCGA). Correlations and p-value based on Pearson correlation coefficient. **D.** Consensus transcriptional molecular classification of tumors in the TCGA Bladder Cancer (TCGA-BLCA) cohort stratified based on *ERBB2* status. Ba/Sq: basal squamous, LumNS: luminal nonspecified, LumP: luminal papillary, LumU: luminal unstable, NE-like: neuroendocrine-like**. E.** Immune cell deconvolution analysis of mRNA expression data from the TCGA-BLCA cohort. The size of the circles reflects the p-value of enriched immune cells in either the *ERBB2* amplified or *ERBB2* mutated samples compared to WT (two-sided paired sample Wilcoxon test). Raw data and p-values for A-E are provided in Source Data.

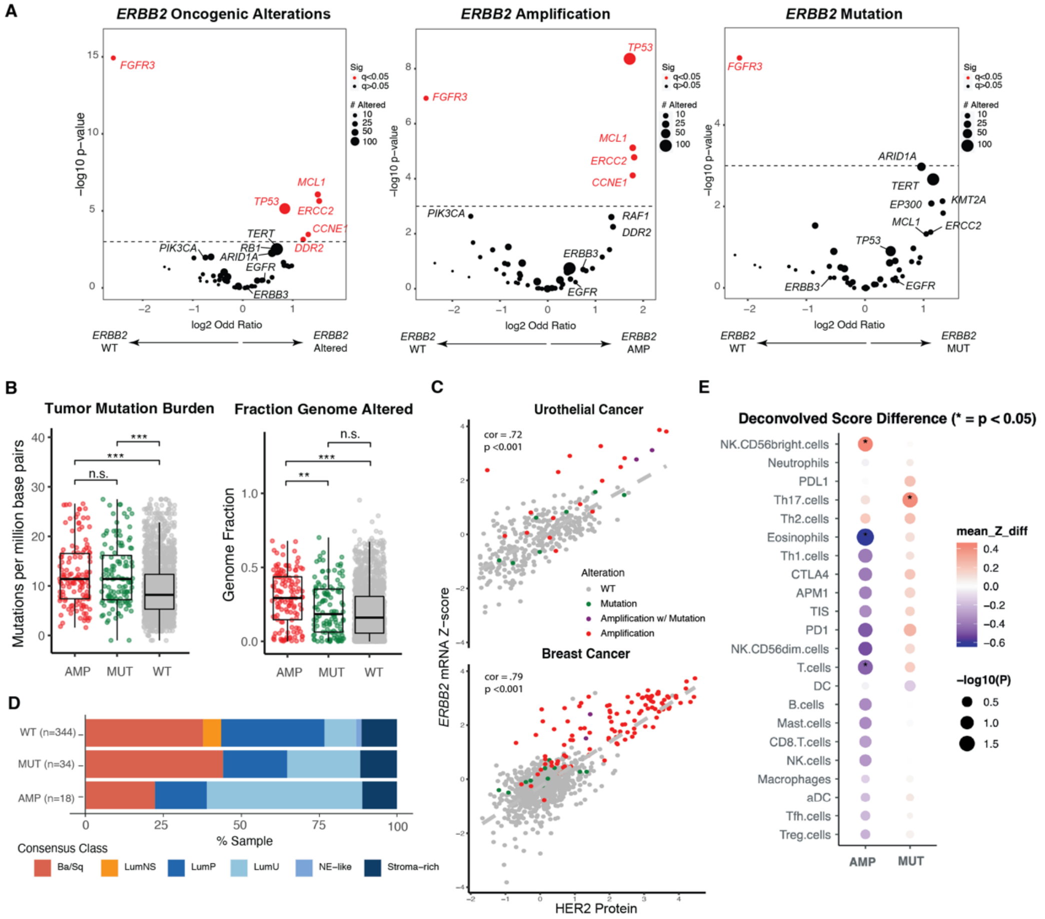

Figure S4. Genomic and molecular profiling of patient-matched tumors and xenograft-organoid models. A. Whole exome sequencing-based single-base pair mutational signature analysis of 4 urothelial cancer organoid models and the patient-matched urothelial cancers from which they were derived. Annotation based on the COSMIC signature lists. **B.** Representative immunohistochemistry (IHC) staining of the patient tumors from which the SMBO-170 and SMBO-106 organoids were derived (top); Fluorescence In Situ Hybridization (FISH) of patient tumors (middle) from which the SMBO-170 and SMBO-106 organoid lines were derived, and corresponding FISH staining of the corresponding organoid (bottom). All tumors were urothelial carcinoma, NOS (not otherwise specified). SMBO-170 demonstrated diffuse and strong membranous HER2 expression (3+) and *ERBB2* amplification (10.2-fold based on FISH). SMBO-106 had weak incomplete membranous HER2 expression (1+) and low level *ERBB2* amplification (2.0-fold change).

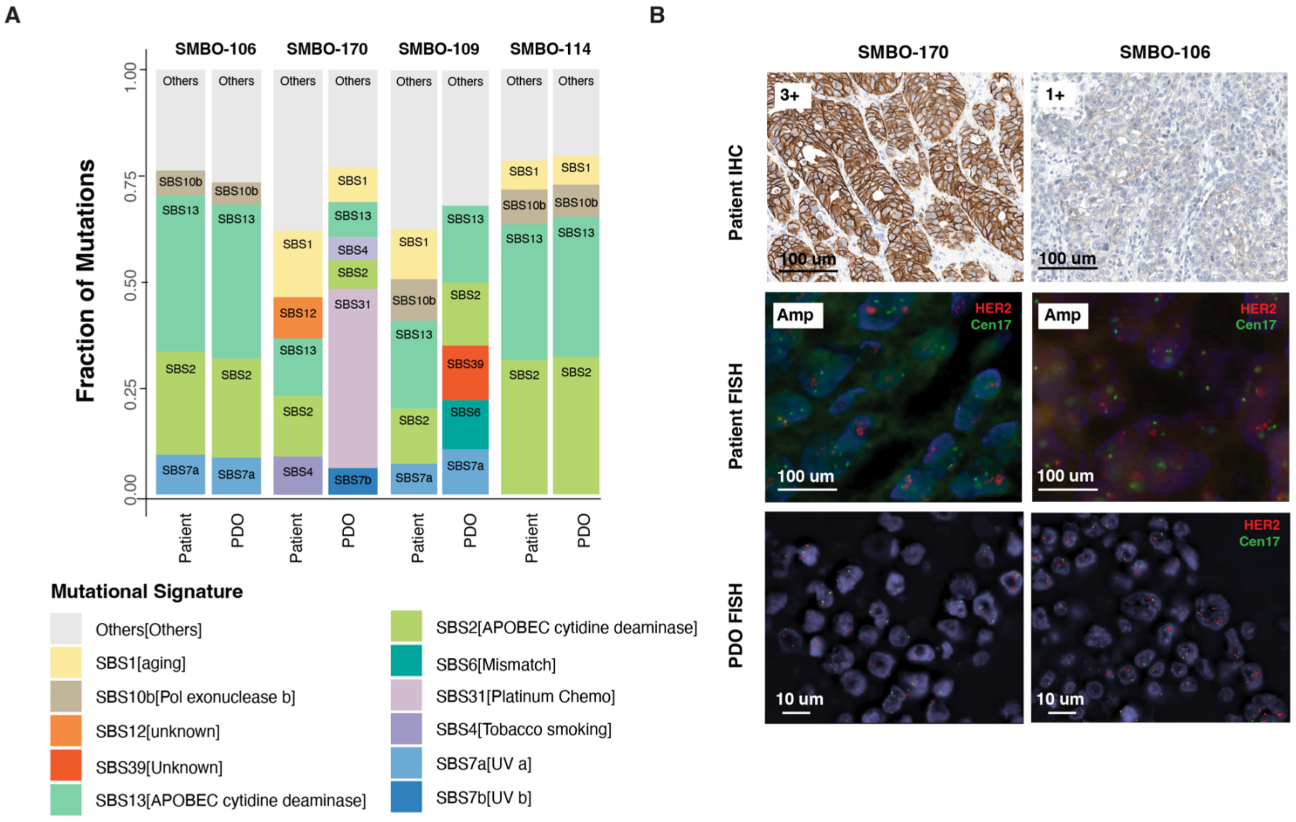

Figure S5. Single-cell DNA sequencing analysis of patient-derived organoids (PDOs). MEDICC2 analysis of (**A**) SMBO-170, (**B**) SMBO-114, and (**C**) SCBO-8 patient derived organoids based on the inferred copy number phylogenies from single-cell DNA sequencing data. The heatmap on the right represents genome wide copy number state profiles for individual cells. **D.** *TP53* and *RB1* copy number density distribution in selected clusters with more than 25 cells identified by k-means clustering. The x-axis represents the raw copy number of each gene.

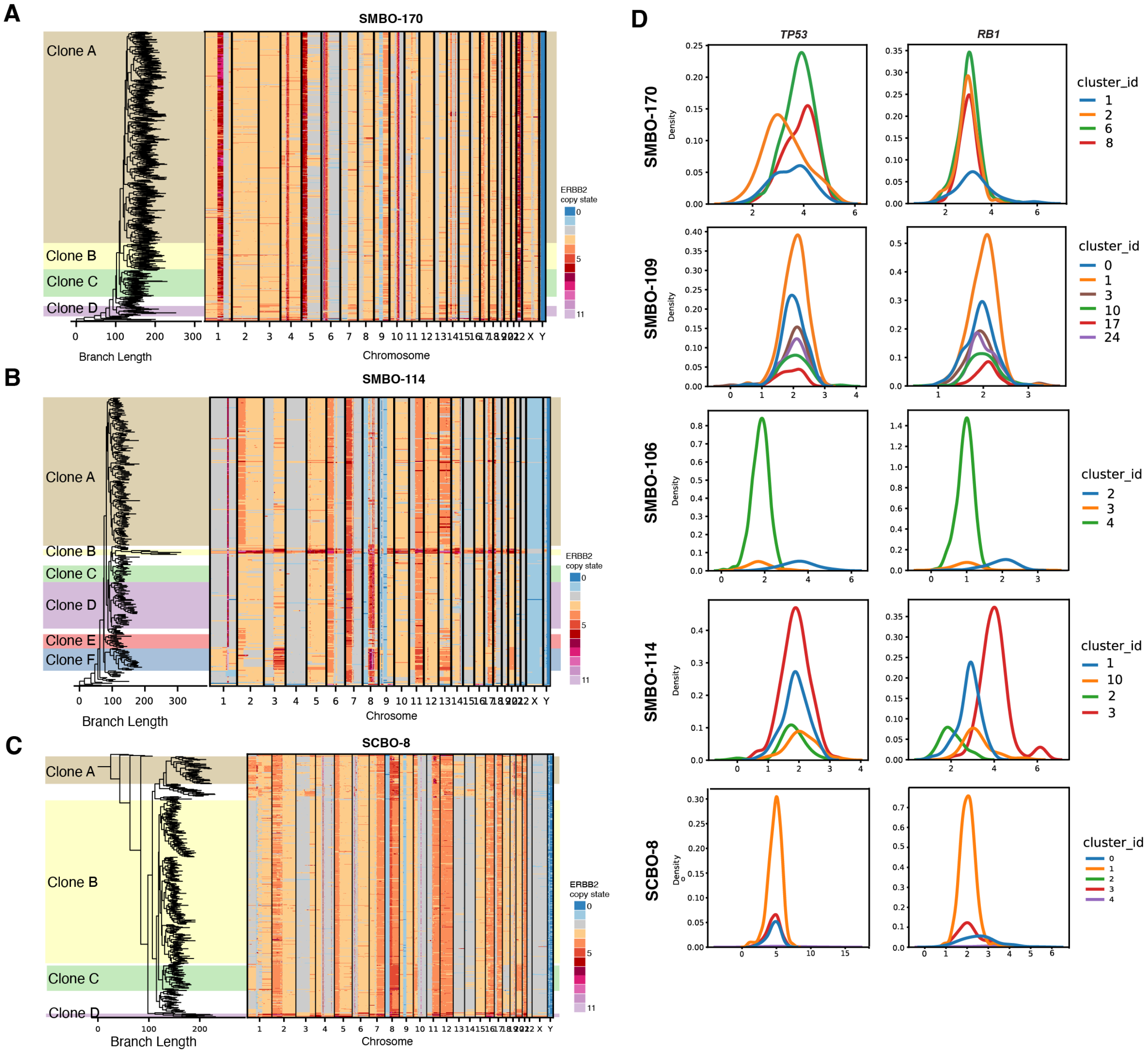

Figure S6. Distribution of expression of *EGFR* and *ERBB2* at the single cell level in urothelial cancer patient derived organoid models. UMAP plots of *ERBB2* (green) and *EGFR* (magenta) expression at single cell resolution. Cells expressing high *ERBB2* and high *EGFR* are colored in grey. High expression is defined as expression level greater than the third quartile within each sample. Raw data are provided in Source Data.

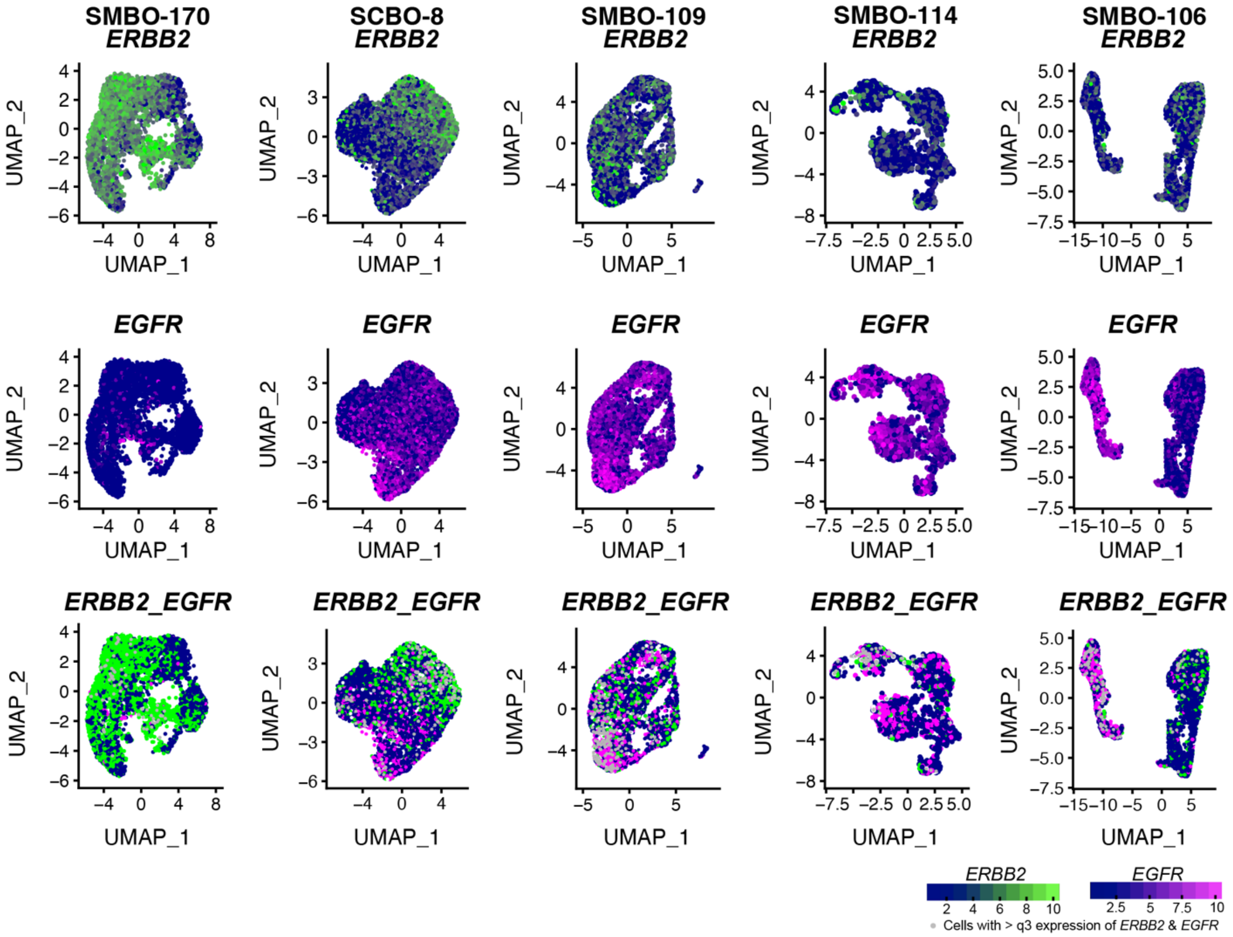

Figure S7. Sensitivity of patient-derived bladder cancer xenografts and organoids to trastuzumab deruxtecan (T-DXd) and the HER kinase inhibitor neratinib. A. SMBO-106 and SMBO-170 organoids growing in matrigel (3D) and 6-well plates (2D) were treated with neratinib at the concentrations indicated for 1 hour. Data are representative of three independent experiments. **B.** SMBO-170, SMBO-106, SMBO-114 and SMBO-9 urothelial cancer organoids and BT474 breast cancer cells were treated with neratinib (100nM) or DMSO as control. Cells were collected after 48 of treatment with neratinib. Bar plots represent the fraction of cells in each phase of the cell cycle as determined by flow cytometry. Results are shown as means ± standard deviation (SD) for three independent experiments. **C.** Mice with established SMBO-170 and SMBO-106 xenografts were treated with neratinib (20 mg/kg; p.o. QD×5, blue), T-DXd (10 mg/kg, i.v., once every 3 weeks for 9 weeks, red) or their vehicles as control. Two-way ANOVA test (Prism) was used for statistical analysis without adjustment. SMBO-170: p<0.001 vehicle vs neratinib; p<0.001 vehicle vs T-DXd; SMBO-106: P<0.001 vehicle vs neratinib; p<0.001 vehicle vs T-DXd. Results are shown as spider plots, with each line representing an individual xenograft-bearing mouse. **D.** Mice with established SMBO-106 xenografts were treated with T-DXd after the tumors reached a mean volume of 500 mm^3. Complete responses were observed in all 8 T-DXd treated mice. After 3 doses of treatment, mice were monitored for 4 months with no evidence of tumor recurrence observed. **E.** Fraction of SMBO-170, SMBO-106 and BT474 cells in each phase of cell cycle 48 hours after exatecan (100nM) treatment. Data presented as mean values ± SD for three independent experiments. Source data for B-G are provided as a Source Data file. An example of the gating strategy for B and E is provided in **Figure S9** in Supplementary Information file.

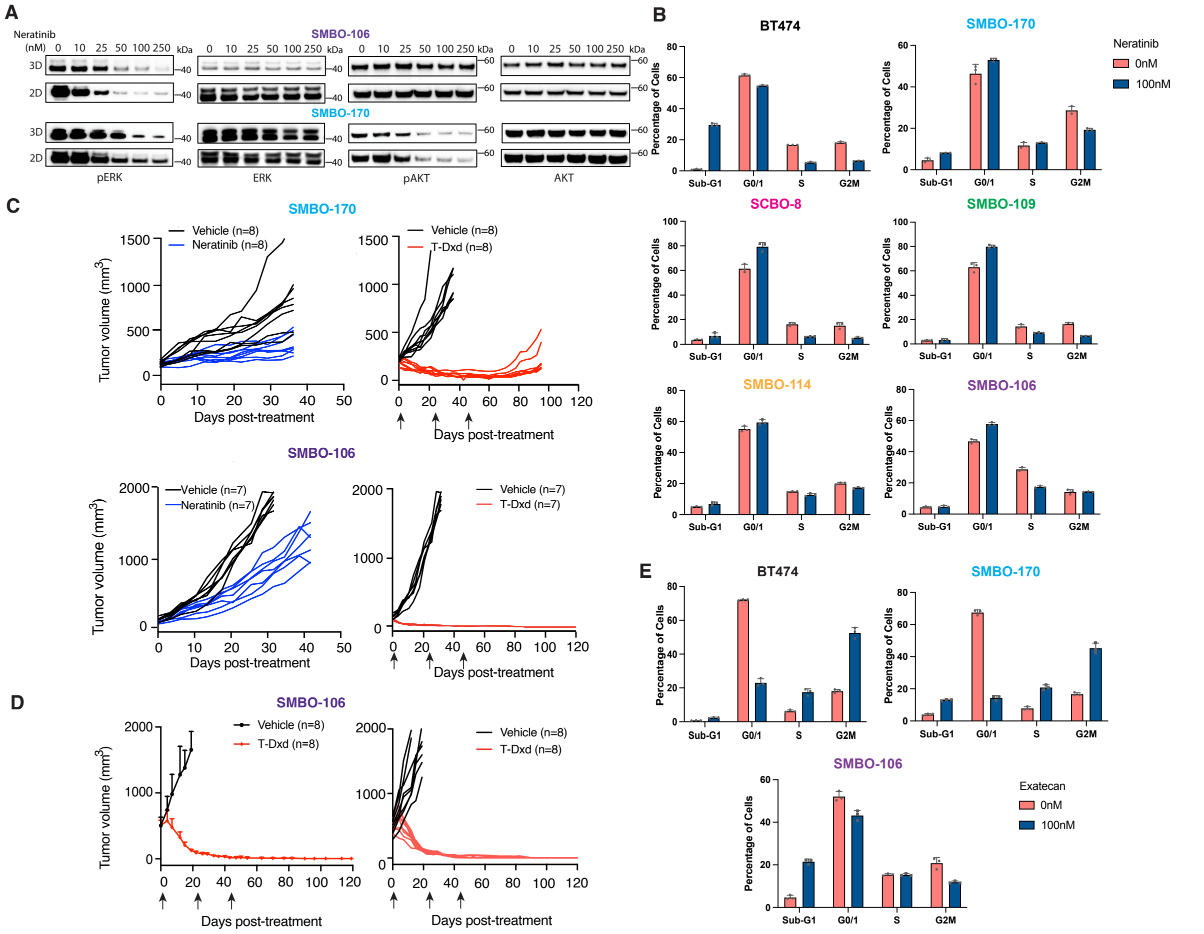

Figure S8. Comparison of sensitivity of urothelial cancer patient-derived xenografts to trastuzumab deruxtecan (T-DXd) and enfortumab vedotin (EV). A/B. Mice with established SMBO-170 (**A**) and SMBO-106 (**B**) xenografts were treated with T-DXd (10 mg/kg, i.v., once every 3 weeks for 9 weeks, red), EV (5 mg/kg, i.v., on day 8, 11, 14, orange) or their vehicles only as control. SMBO-170: p<0.001 vehicle vs T-DXd; p<0.001 vehicle vs EV; SMBO-106: p<0.001 vehicle vs T-DXd; SMBO-106 p<0.001 vehicle vs EV. Two-way ANOVA test (Prism) was used for statistical analysis without adjustment. Data are presented as mean values ± SD. Only the upper error bars are displayed for clarity. **C**. Sensitivity of 15 urothelial organoids to exatecan, as measured by IC50 values calculated using GraphPad Prism. Source data for A-C are provided as a Source Data file.

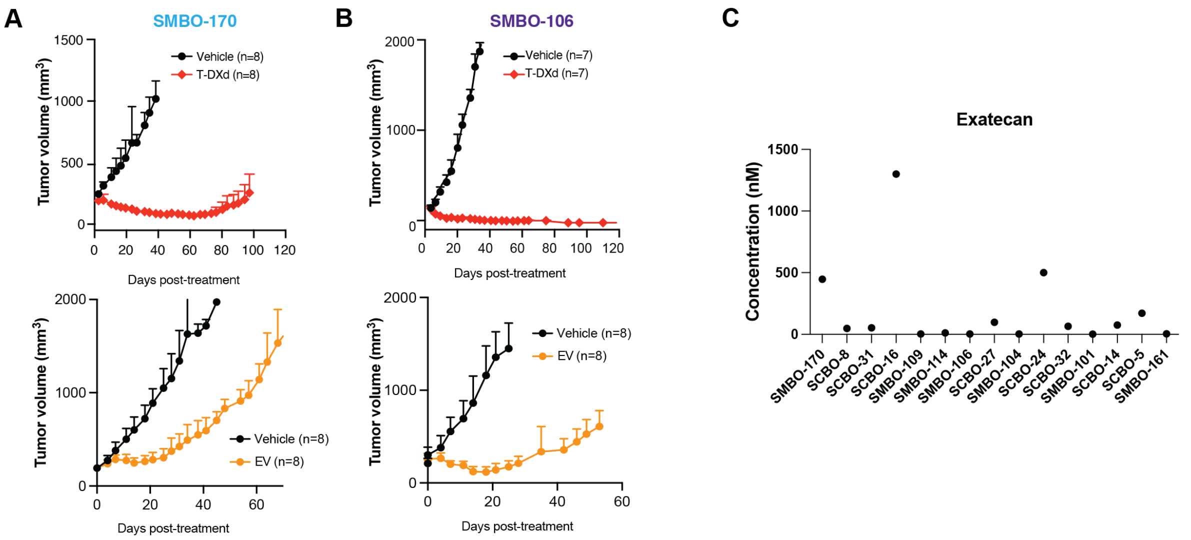

Supplementary Table 1. Summary of 45 urothelial cancer patient-derived xenograft/organoid lines and the corresponding clinical data from the patients from which they were derived. 35 models were derived from patients with a bladder primary site and 10 from patients with an upper tract urothelial cancer (UTUC) primary site. 42 of the models were derived from tumor tissue collected from the primary tumor site, and 3 from metastatic sites.

Sample	Primary/Met	Upper Tract / Bladder Cancer	Stage	Grade	Pathologic Classification	*ERBB2* Alteration	Sex
**SMBO-101**	Met-Lung	Bladder	T3	High	UC with mixed histology features including NOS, squamous and focal sarcomatoid	WT	M
**SMBO-104**	Primary	Bladder	T2	High	Invasive UC-NOS	WT	F
**SMBO-106**	Primary	Bladder	T2	High	Invasive UC-NOS	Amplification	F
**SMBO-109**	Primary	Bladder	T2	High	UC NOS with nested/glandular features	WT	F
**SMBO-114**	Primary	UTUC	T3/N2	High	UC-NOS	Mutation	M
**SMBO-170**	Primary	Bladder	M1	High	Invasive UC-NOS	Amplification	F
**SCBO-1**	Primary	Bladder	Ta	High	Papillary UC, non-invasive	WT	F
**SCBO-2**	Primary	Bladder	T1	High	Invasive UC-NOS	WT	M
**SCBO-3**	Primary	Bladder	T1	Low/High	Invasive UC-NOS	WT	M
**SCBO-4**	Primary	Bladder	T2	High	Invasive UC-NOS	Amplification	F
**SCBO-5**	Primary	Bladder	T1+CIS	High	Invasive UC-NOS	Amplification, mutation	M
**SCBO-6**	Primary	Bladder	T1+CIS	High	Invasive UC-NOS	WT	M
**SCBO-7**	Primary	Bladder	Ta	Low	Papillary UC, non-invasive	WT	F
**SCBO-8**	Primary	Bladder	Ta	High	Papillary UC, non-invasive	Amplification, mutation	M
**SCBO-9**	Primary	Bladder	T1	Low/High	Invasive UC-NOS	WT	F
**SCBO-10**	Primary	Bladder	T2	High	Invasive UC-NOS	WT	M
**SCBO-11**	Primary	Bladder	T1+CIS	Low	Squamous cell carcinoma	WT	M
**SCBO-12**	Primary	Bladder	Ta	Low	Papillary UC, non-invasive	WT	M
**SCBO-13**	Primary	Bladder	T1+CIS	High	UC, non-invasive	Mutation	M
**SCBO-15**	Primary	Bladder	T3	High	Invasive UC-NOS	WT	F
**SCBO-16**	Primary	Bladder	Ta	Low	Papillary UC, non-invasive	WT	M
**SCBO-19**	Primary	Bladder	T1	High	Invasive UC-NOS	WT	M
**SCBO-24**	Primary	Bladder	T3	High	Invasive UC with micropapillary	WT	F
**SCBO-25**	Primary	Bladder	T4	High	Invasive UC with nested	WT	M
**SCBO-27**	Primary	Bladder	T1	High	Invasive UC-NOS	Mutation	M
**SCBO-28**	Primary	Bladder	T2	High	Invasive UC-NOS	Mutation	F
**SCBO-29**	Primary	Bladder	T3	High	Invasive UC-NOS	WT	M
**SCBO-30**	Primary	Bladder	Ta	Low/High	Papillary UC, non-invasive	WT	F
**SCBO-31**	Primary	Bladder	T2	High	Invasive UC with Squamous Differentiation	Amplification	F
**SCBO-33**	Primary	Bladder	T1	Low/High	Invasive UC-NOS	WT	M
**SCBO-37**	Primary	Bladder	T2	High	Invasive UC-NOS	WT	M

**SCBO-38**	Primary	Bladder	T1	High	Invasive UC-NOS	Amplification	M
**SCBO-39**	Primary	Bladder	T2	High	Invasive UC with Squamous Differentiation	WT	F
**SCBO-43**	Primary	Bladder	T4	High	Clear Cell Adenocarcinoma	Amplification	F
**SCBO-46**	Met-LN	Bladder	N1	High	Metastatic Lymph Node with nested and micropapillary	WT	M
**SCBO-49**	Primary	Bladder	T2	High	Invasive UC with Squamous Differentiation and Neuroendocrine	WT	F
**SMBO-201**	Primary	UTUC	pT2/pN0	High	UC-NOS	WT	M
**SMBO-203**	Met-abdominal	UTUC	pTx/pNx	High	UC-NOS	WT	M
**SMBO-205**	Primary	UTUC	pTa	High	UC-NOS	WT	F
**SMBO-209**	Primary	UTUC	pT3/pN1	High	UC-NOS	WT	M
**SMBO-219**	Primary	UTUC	pT3/pNx	High	UC-NOS	WT	F
**SMBO-230**	Primary	UTUC	pTa/pN0	Low	UC-NOS	WT	F
**SMBO-232**	Primary	UTUC	pT3/pN2	High	UC-NOS	WT	F
**SMBO-240**	Primary	UTUC	pT2/pN0	High	UC-NOS	WT	M
**SMBO-247**	Primary	UTUC	pTa/pN0	High	UC-NOS	WT	M

Abbreviations: UTUC, upper tract urothelial carcinoma; CIS, carcinoma *in situ*; UC-NOS, urothelial carcinoma-not otherwise specified; LN, lymph node; WT, wildtype.

Figure S9. **Representative flow cytometry gating strategy for cell cycle analysis.** Representative example of the flow cytometry gating strategy used to determine cell cycle distribution based on propidium iodide (PI) staining. Cells were first gated to exclude debris and doublets, followed by analysis of DNA content (PI-A) to quantify the percentage of cells in sub-G1, G0/G1, S, and G2/M phases.

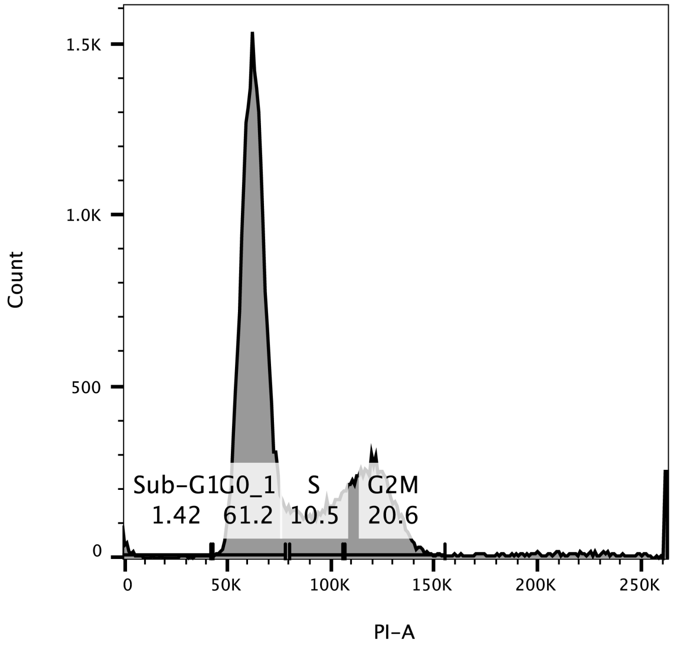

Supplement: Supplementary file 1 — Supplementary Information [file 41467_2025_67643_MOESM1_ESM.pdf]
