# Supplementary material for: Determinants of sensitivity to HER2-targeted antibody drug conjugates in urothelial cancer
Source: Nat Commun. 2025 Dec 20;17:919. doi: 10.1038/s41467-025-67643-2 (PMC12830848; doi:10.1038/s41467-025-67643-2)
Supplement: Supplementary file 2 — Reporting Summary [file 41467_2025_67643_MOESM2_ESM.pdf]

nature portfolio

Corresponding author(s): David B. Solit

Last updated by author(s): Oct 9, 2025

Reporting Summary

Nature Portfolio wishes to improve the reproducibility of the work that we publish. This form provides structure for consistency and transparency in reporting. For further information on Nature Portfolio policies, see our Editorial Policies and the Editorial Policy Checklist.

Statistics

For all statistical analyses, confirm that the following items are present in the figure legend, table legend, main text, or Methods section.

n/a	Confirmed	
☐	☒	The exact sample size (*n*) for each experimental group/condition, given as a discrete number and unit of measurement
☐	☒	A statement on whether measurements were taken from distinct samples or whether the same sample was measured repeatedly
☐	☒	The statistical test(s) used AND whether they are one- or two-sided<br>*Only common tests should be described solely by name; describe more complex techniques in the Methods section.*
☒	☐	A description of all covariates tested
☐	☒	A description of any assumptions or corrections, such as tests of normality and adjustment for multiple comparisons
☐	☒	A full description of the statistical parameters including central tendency (e.g. means) or other basic estimates (e.g. regression coefficient) AND variation (e.g. standard deviation) or associated estimates of uncertainty (e.g. confidence intervals)
☐	☒	For null hypothesis testing, the test statistic (e.g. F, t, r) with confidence intervals, effect sizes, degrees of freedom and P value noted<br>*Give P values as exact values whenever suitable.*
☒	☐	For Bayesian analysis, information on the choice of priors and Markov chain Monte Carlo settings
☒	☐	For hierarchical and complex designs, identification of the appropriate level for tests and full reporting of outcomes
☐	☒	Estimates of effect sizes (e.g. Cohen's *d*, Pearson's *r*), indicating how they were calculated

Our web collection on statistics for biologists contains articles on many of the points above.

Software and code

Policy information about availability of computer code

Data collection	Please see our methods section and data availability section in the manuscript.
Data analysis	Please see our methods section and data availability section in the manuscript.

For manuscripts utilizing custom algorithms or software that are central to the research but not yet described in published literature, software must be made available to editors and reviewers. We strongly encourage code deposition in a community repository (e.g. GitHub). See the Nature Portfolio guidelines for submitting code & software for further information.

Data

Policy information about availability of data

All manuscripts must include a data availability statement. This statement should provide the following information, where applicable:

- Accession codes, unique identifiers, or web links for publicly available datasets
- A description of any restrictions on data availability
- For clinical datasets or third party data, please ensure that the statement adheres to our policy

Please see our data availability section in the manuscript.

Research involving human participants, their data, or biological material

Policy information about studies with human participants or human data. See also policy information about sex, gender (identity/presentation), and sexual orientation and race, ethnicity and racism.

Reporting on sex and gender	We compared the correlation of ERBB2 alteration with patient demographic data including sex, race, and smoking status in patients with urothelial cancer. The cohort included 1,457 male and 572 female patients.
Reporting on race, ethnicity, or other socially relevant groupings	We compared the correlation of ERBB2 alteration with patient demographic data including sex, race, and smoking status in patients with urothelial cancer. By race, the cohort included 71 White, 84 Black, 71 Asian, and 32 patients of Other racial backgrounds. Smoking status was categorized as 378 Never smokers, 513 Former smokers, and 109 Active smokers.
Population characteristics	The detailed information are accessible via cBioPortal. Detailed information on patient-derived organoids and xenografts and the corresponding clinical data from the patients from which they were derived are provided in Sumpplementary Table 1.
Recruitment	We analyzed MSK-IMPACT (Memorial Sloan Kettering - Integrated Mutation Profiling of Actionable Cancer Targets) data for 56,801 unique tumor samples analyzed as part of an institution-wide prospective sequencing study (ClinicalTrials.gov identifier: NCT01775072) at Memorial Sloan Kettering Cancer Center (New York, NY).
Ethics oversight	For generation of patients derived models, patients were consented to MSKCC IRB 06-107, 12-245, or 19-105 or Columbia University Medical Center AAAN8850 or AAAT2128.

Note that full information on the approval of the study protocol must also be provided in the manuscript.

Field-specific reporting

Please select the one below that is the best fit for your research. If you are not sure, read the appropriate sections before making your selection.

[x] Life sciences [ ] Behavioural & social sciences [ ] Ecological, evolutionary & environmental sciences

For a reference copy of the document with all sections, see nature.com/documents/nr-reporting-summary-flat.pdf

Life sciences study design

All studies must disclose on these points even when the disclosure is negative.

Sample size	No statistical method was used to predetermine sample size. We collected the patient data available who has gone through MSK-IMPACT study. For patient cohort, an estimated tumor purity of less than 20% and those with a tumor mutational burden exceeding the 95th percentile of their respective cancer type were excluded. The final cohort was comprised of 42,415 tumors. Sample size was determined according to previous publications where at least 7-8 animals per group were analyzed for in vivo drug testing and 3 replicates per group were analyzed for in vitro drug testing. No data were excluded from the in vivo and in vitro analysis.
Data exclusions	For patient cohort, an estimated tumor purity of less than 20% and those with a tumor mutational burden exceeding the 95th percentile of their respective cancer type were excluded.
Replication	For SMBO-106 PDX treated with T-DXd, we repeated this experiment with larger tumor size and confirmed the durable responses. For in vitro testing, at least 3 independent experiments were performed.
Randomization	The samples were randomly designated into experimental groups.
Blinding	The investigators were blinded to group allocation during data collection and analysis process.

Reporting for specific materials, systems and methods

We require information from authors about some types of materials, experimental systems and methods used in many studies. Here, indicate whether each material, system or method listed is relevant to your study. If you are not sure if a list item applies to your research, read the appropriate section before selecting a response.

Materials & experimental systems

n/a	Involved in the study	
	x	Antibodies
	x	Eukaryotic cell lines
x		Palaeontology and archaeology
	x	Animals and other organisms
	x	Clinical data
x		Dual use research of concern
x		Plants

Methods

n/a	Involved in the study	
x		ChIP-seq
	x	Flow cytometry
x		MRI-based neuroimaging

Antibodies

Antibodies used

Primary antibodies (1:1000 dilution in TBST/3% BSA) from Cell Signaling Technology: HER2 (#4290), EGFR (Y1173) (#2232), HER3 (#12708), ERK (#9102), p-ERK (T202/Y204) (#9101), AKT (#9272), p-AKT (Ser473) (#9271), Rb (4H1) (#9309), GAPDH (#2118), GATA3 (#5852), CD44 (#37259), KRT14 (#30731) and antibody from Abcam: KRT5 (#52635) and Nectin-4 (#155692). HER2/neu IHC antibody (1:10 dilution): Ventana #790-2991

Validation

All antibodies utilized in this research were acquired from commercial suppliers, and their validation statements can be found on the websites, which provide detailed validation and associated reference publications.
HER2 (#4290): https://www.cellsignal.com/products/primary-antibodies/her2-erbb2-d8f12-xp-rabbit-mab/4290?srsltid=AfmBOop1h7FwlCYrBT2osE62eU0xapCxdKbBJkScb2Xqo6aSJZAgF773
EGFR (Y1173) (#2232): https://www.cellsignal.com/products/primary-antibodies/egf-receptor-antibody/2232
HER3 (#12708): https://www.cellsignal.com/products/primary-antibodies/her3-erbb3-d22c5-xp-rabbit-mab/12708
ERK (#9102): https://www.cellsignal.com/products/primary-antibodies/p44-42-mapk-erk1-2-antibody/9102
p-ERK (T202/Y204) (#9101): https://www.cellsignal.com/products/primary-antibodies/phospho-p44-42-mapk-erk1-2-thr202-tyr204-antibody/9101
AKT (#9272): https://www.cellsignal.com/products/primary-antibodies/akt-antibody/9272
p-AKT (Ser473) (#9271): https://www.cellsignal.com/products/primary-antibodies/phospho-akt-ser473-antibody/9271
Rb (4H1) (#9309): https://www.cellsignal.com/products/primary-antibodies/rb-4h1-mouse-mab/9309
GAPDH (#2118): https://www.cellsignal.com/products/primary-antibodies/gapdh-14c10-rabbit-mab/2118
GATA3 (#5852): https://www.cellsignal.com/products/primary-antibodies/gata-3-d13c9-xp-rabbit-mab/5852
KRT5 (#52635):https://www.abcam.com/en-us/products/primary-antibodies/cytokeratin-5-antibody-ep1601y-cytoskeleton-marker-ab52635
CD44 (#37259): https://www.cellsignal.com/products/primary-antibodies/cd44-e7k2y-xp-rabbit-mab/37259?srsltid=AfmBOopNKB-dylOZ1pvq--2Zy9pno0W-DwJTsm4iRRfUoJoyEybtrS9A
KRT14 (#30731): https://www.cellsignal.com/products/primary-antibodies/keratin-14-e7j5w-rabbit-mab/30731
Nectin-4 (#155692): https://www.abcam.com/en-us/products/primary-antibodies/nectin-4-antibody-ab155692?srsltid=AfmBOooyKPISUESr1KivV5VnSG1Xaa2XmuTo895fzXAStOEc2zA0j1Pu
HER2 (IHC, Ventana #790-2991): https://diagnostics.roche.com/us/en/products/lab/her-2-neu-4b5-pathway-rtd001084.html

Eukaryotic cell lines

Policy information about cell lines and Sex and Gender in Research

Cell line source(s)	BT474 (ATCC, HTB-20), and detailed information (sex, pathologic classification and disease stage) of patient-derived organoids and xenografts are available in Supplementary Table 1.
Authentication	No authentication was performed.
Mycoplasma contamination	The cells were tested negative for mycoplasma contamination.
Commonly misidentified lines (See ICLAC register)	This study does not involve commonly misidentified cell lines.

Animals and other research organisms

Policy information about studies involving animals; ARRIVE guidelines recommended for reporting animal research, and Sex and Gender in Research

Laboratory animals	Mice: NOD-SCID IL2Rg–/– (NSG ®) mice (The Jackson Laboratory) males 6-8 weeks old. Mice were consistently housed and controlled under the environmental conditions of 21±1.5°C temperature, 55±10% humidity and a 12h light–dark cycle (lights were on from 6:00 to 18:00).
Wild animals	Study did not involve wild animals
Reporting on sex	We used all male mice because bladder cancer arise mostly men (3:1 ratio)

Field-collected samples	The study did not involve field-collected samples.
Ethics oversight	The staff of the Research Animal Resource Center make sure that animals used in research and teaching at Memorial Sloan Kettering (MSK) are well cared for. The Institutional Animal Care and Use Committee (IACUC) oversees MSK's Animal Care and Use Program. MSK IACUC approved the study protocol.

Note that full information on the approval of the study protocol must also be provided in the manuscript.

Clinical data

Policy information about clinical studies
All manuscripts should comply with the ICMJE guidelines for publication of clinical research and a completed CONSORT checklist must be included with all submissions.

Clinical trial registration	Clinical trial registration is not performed since this is a retrospective data analysis.
Study protocol	Patients were consented to MSKCC IRB 06-107, 12-245, or 19-105.
Data collection	To analyze real-world clinical outcomes of patients with metastatic urothelial cancer treated with T-DXd, we identified all patients with urothelial cancer treated with T-DXd at MSKCC prior to January 30th, 2025.
Outcomes	This study does not involve primary and secondary outcomes.

Plants

Seed stocks	*Report on the source of all seed stocks or other plant material used. If applicable, state the seed stock centre and catalogue number. If plant specimens were collected from the field, describe the collection location, date and sampling procedures.*
Novel plant genotypes	*Describe the methods by which all novel plant genotypes were produced. This includes those generated by transgenic approaches, gene editing, chemical/radiation-based mutagenesis and hybridization. For transgenic lines, describe the transformation method, the number of independent lines analyzed and the generation upon which experiments were performed. For gene-edited lines, describe the editor used, the endogenous sequence targeted for editing, the targeting guide RNA sequence (if applicable) and how the editor was applied.*
Authentication	*Describe any authentication procedures for each seed stock used or novel genotype generated. Describe any experiments used to assess the effect of a mutation and, where applicable, how potential secondary effects (e.g. second site T-DNA insertions, mosiacism, off-target gene editing) were examined.*

Flow Cytometry

Plots

Confirm that:

- [ ] The axis labels state the marker and fluorochrome used (e.g. CD4-FITC).
- [ ] The axis scales are clearly visible. Include numbers along axes only for bottom left plot of group (a 'group' is an analysis of identical markers).
- [ ] All plots are contour plots with outliers or pseudocolor plots.
- [ ] A numerical value for number of cells or percentage (with statistics) is provided.

Methodology

Sample preparation	The cells were harvested using 0.25% trypsin, 0.1% EDTA, and resuspended in 10% FBS medium with the collected culture media, washed with ice cold 1% bovine serum albumin PBS (BSA-PBS), fixed with cold (-20C) 1 ml 70% ethanol, stained with Propidium Iodide and then analyzed using an Aria-3 flow cytometer.
Instrument	BD FACSAria-3 Cell Sorter
Software	FlowJo (10.8.1).
Cell population abundance	In a representative analysis, 0.94% of cells were in sub-G1 phase, 70.6% of cells were in G0/G1 phase, 14.8% in S phase, and 12.3% in G2/M phase. Experimental treatments were compared based on shifts in the relative abundance of cells in each phase.
Gating strategy	Debris was excluded by setting a gate on forward scatter area (FSC-A) versus side scatter area (SSC-A). Doublets were excluded by gating on single cells. The Priopidium Iodide intensity histogram was generated and cells were gated into sub-G1, G0/G1, S and G2/M phase.

- [x] Tick this box to confirm that a figure exemplifying the gating strategy is provided in the Supplementary Information.
