# Supplementary material for: Determinants of sensitivity to HER2-targeted antibody drug conjugates in urothelial cancer
Source: Nat Commun. 2025 Dec 20;17:919. doi: 10.1038/s41467-025-67643-2 (PMC12830848; doi:10.1038/s41467-025-67643-2)
Supplement: Supplementary file 4 — Source Data [file 41467_2025_67643_MOESM4_ESM.zip › Source Data/Western uncropped images.docx]

**Figure 2D**

HER2 EGFR


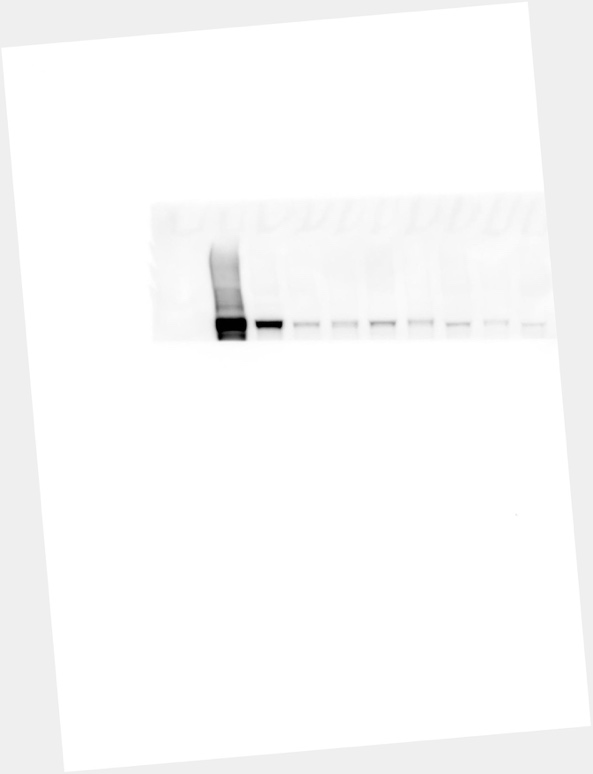

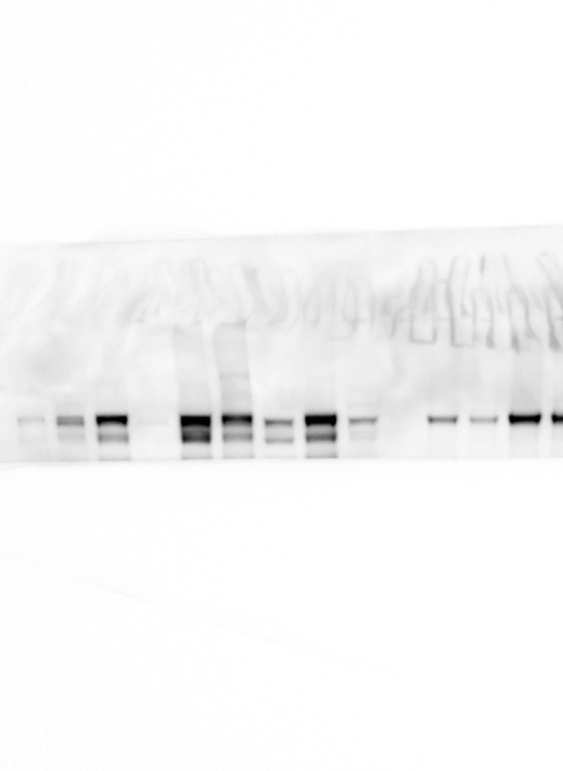


160

260

260

160

HER3 pERK


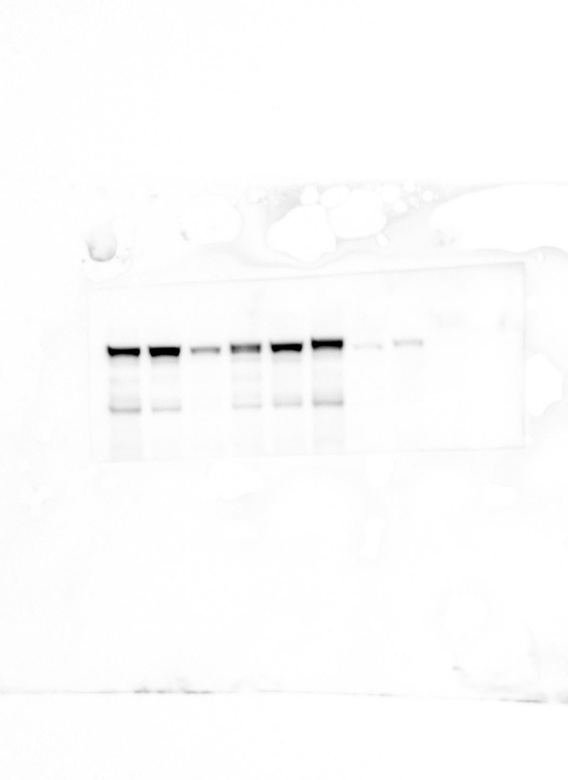

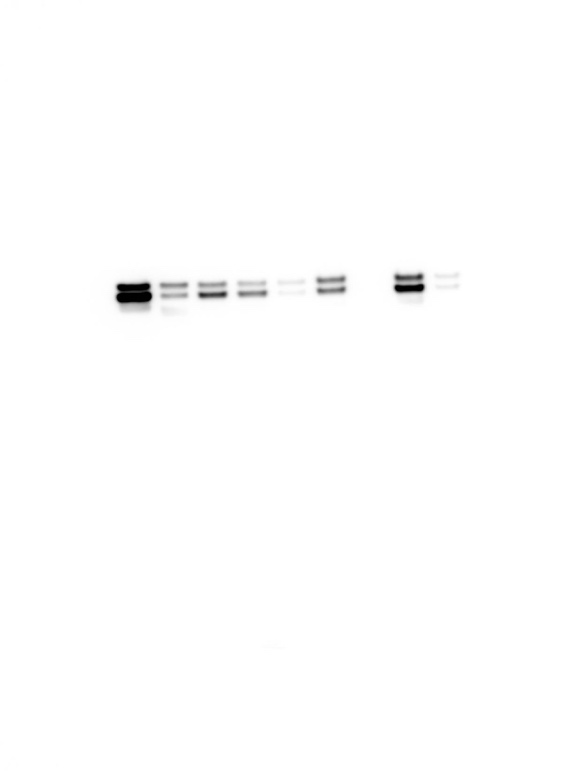


50

40

160

260

ERK pAKT


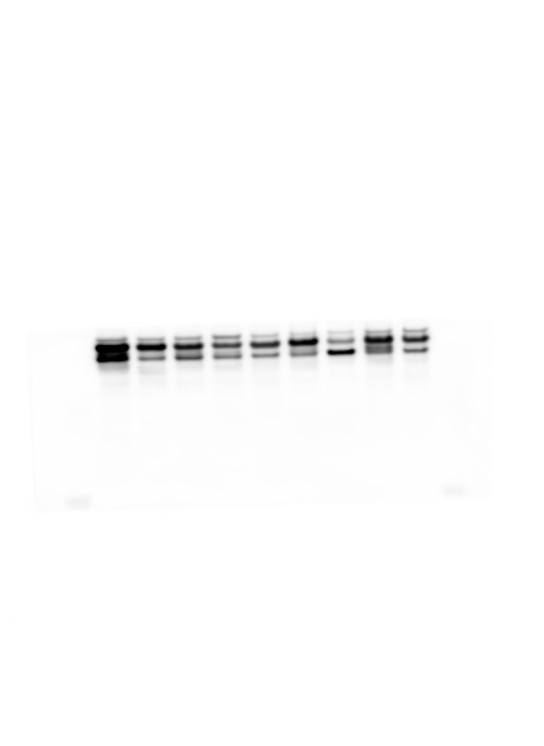

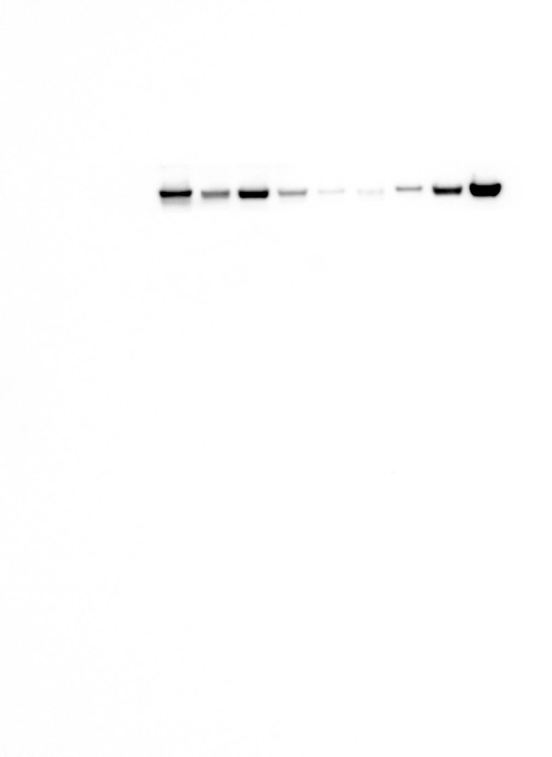


60

50

40

50

AKT RB(4H1)


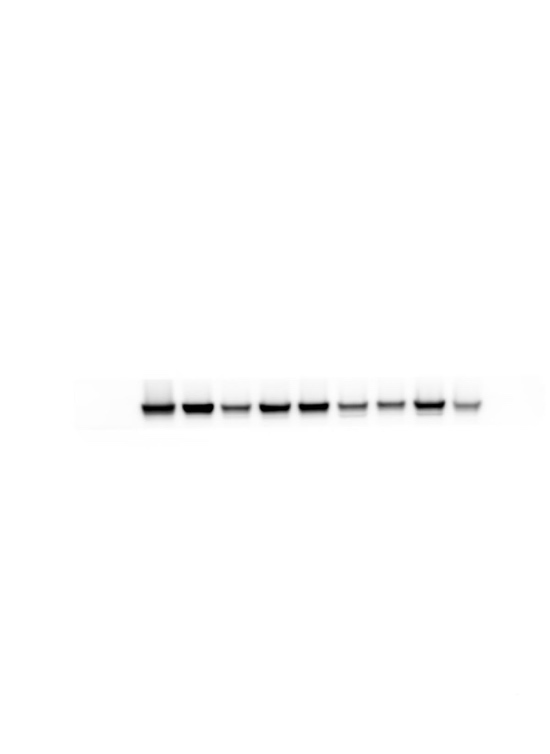

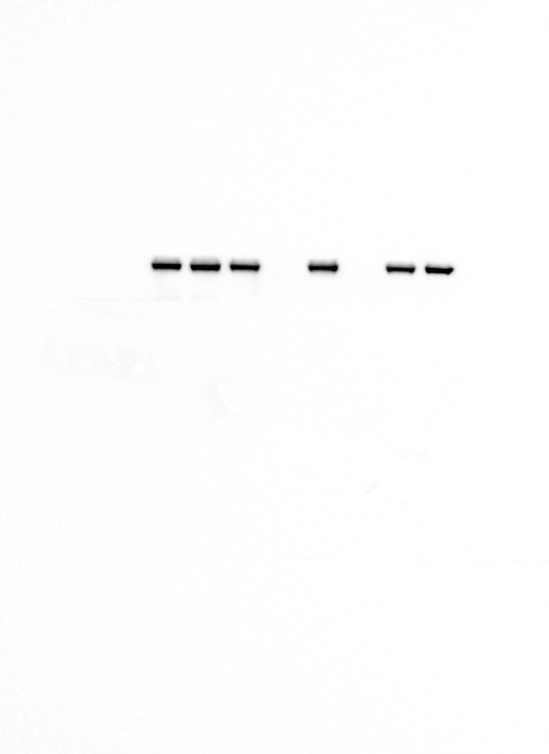


110

60

50

Nectin-4 GATA3


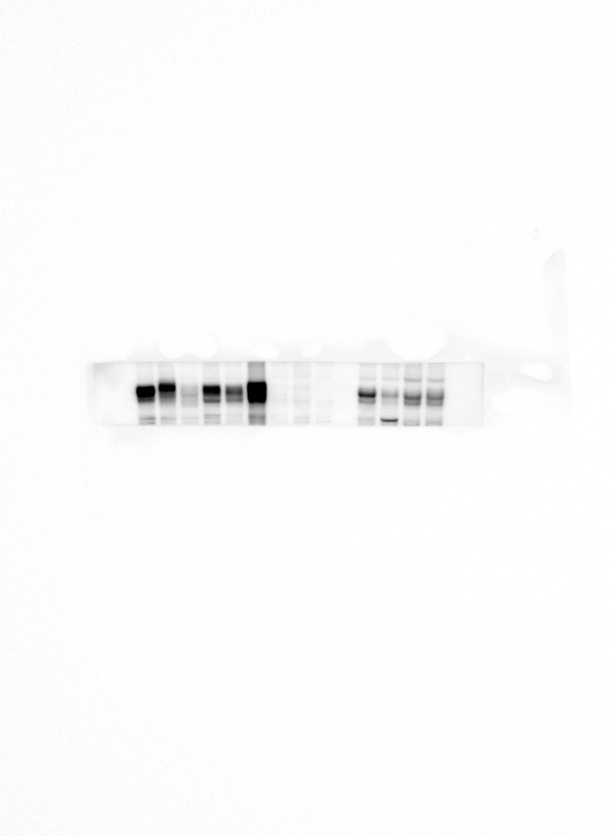

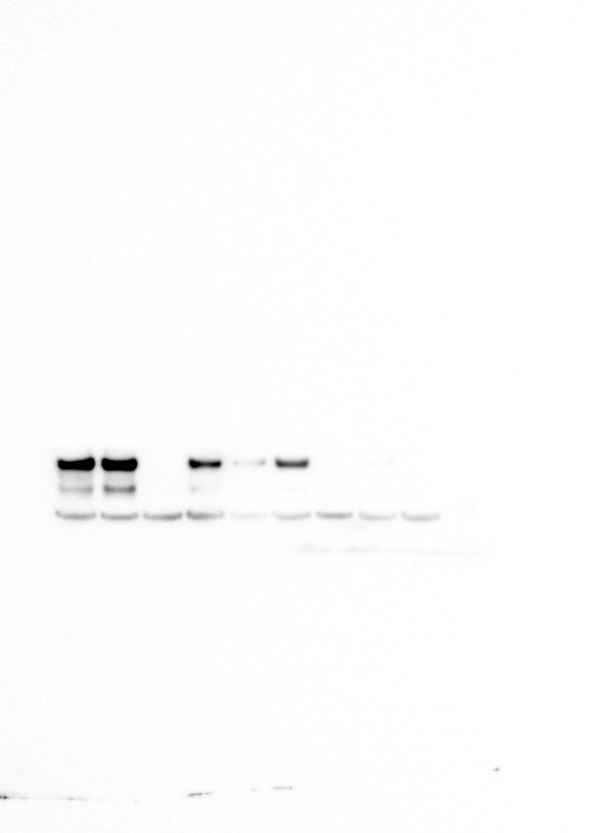


60

40

50

50

60

80

KRT5 CD44


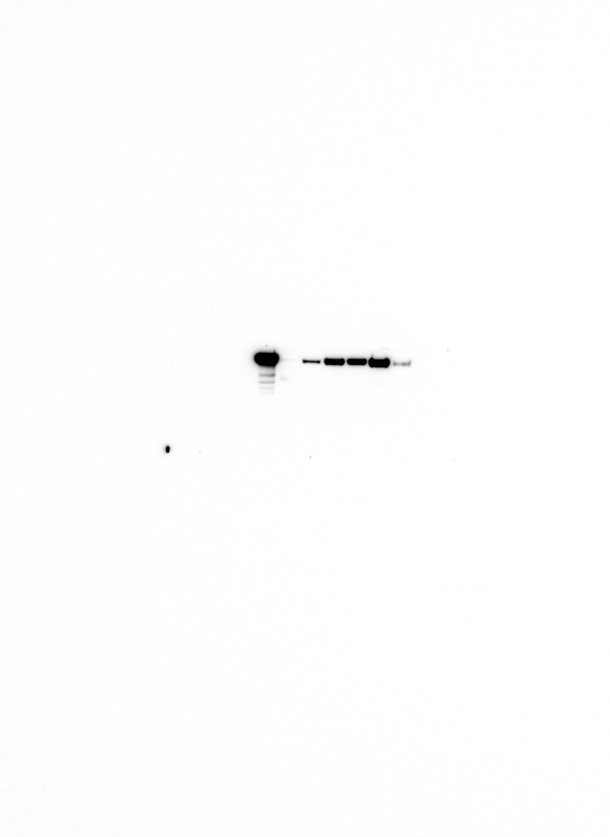

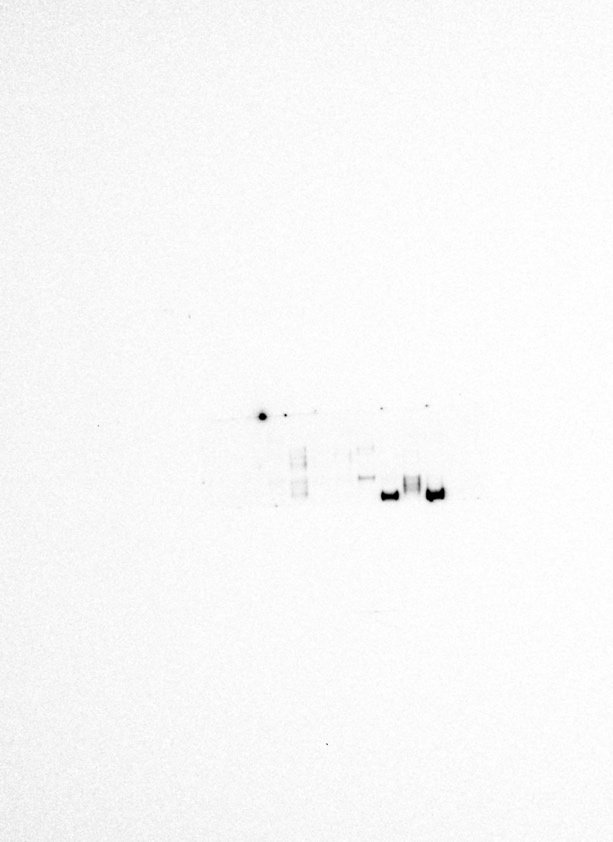


80

110

50

60

KRT14 GAPDH


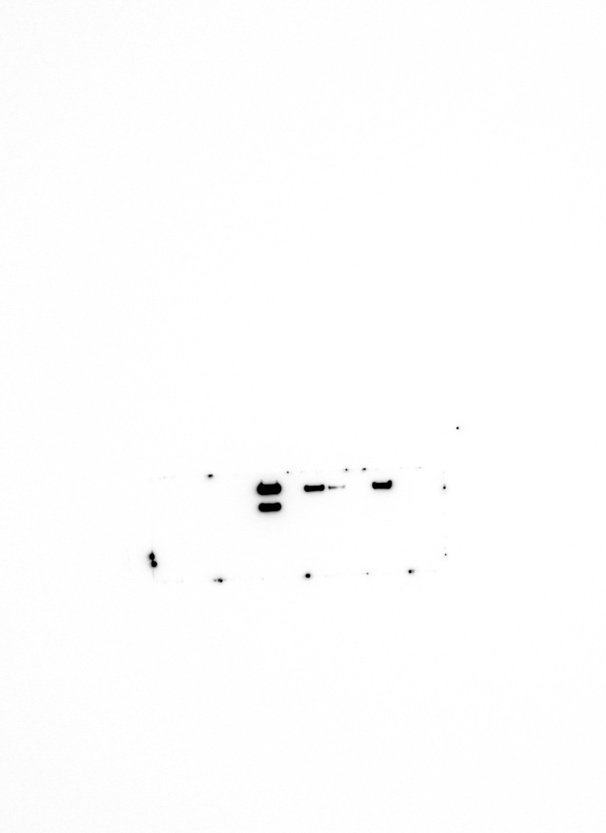

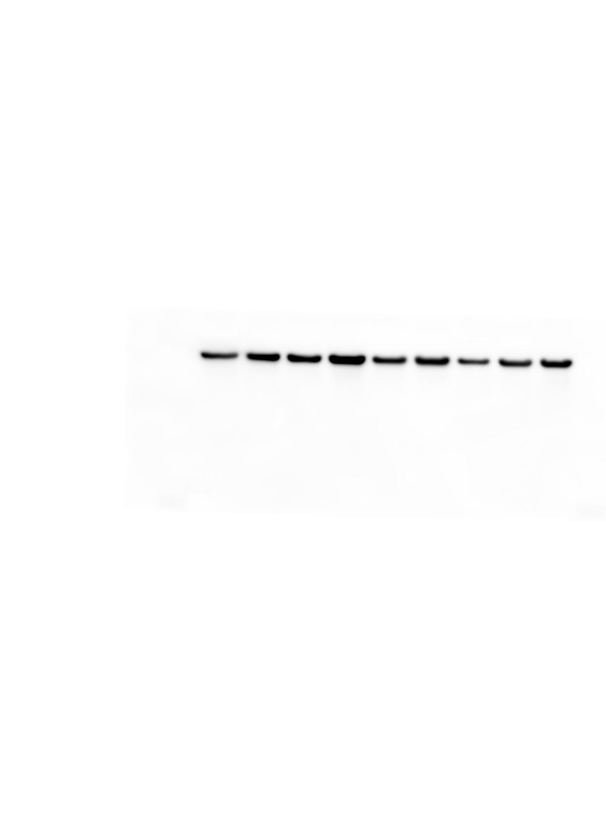


30

40

50

60

**Figure 5A**

BT474

pERK ERK


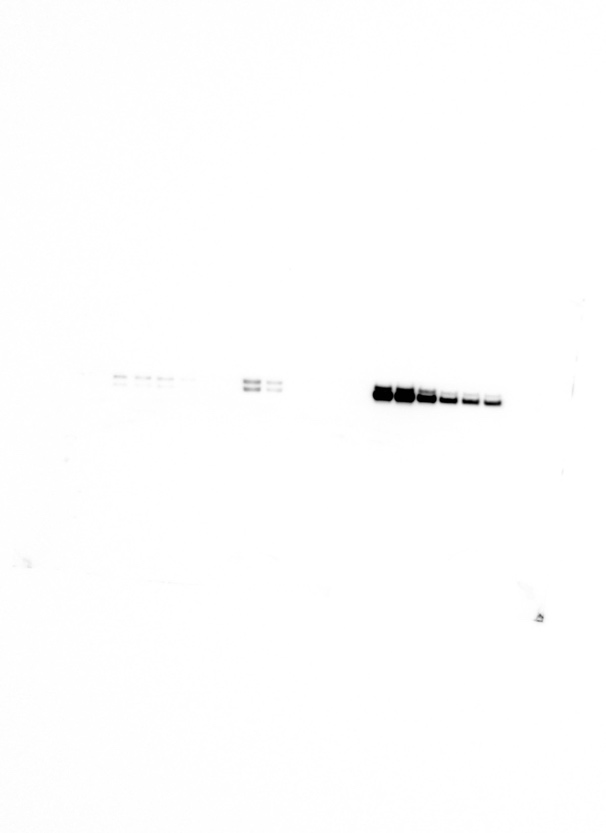

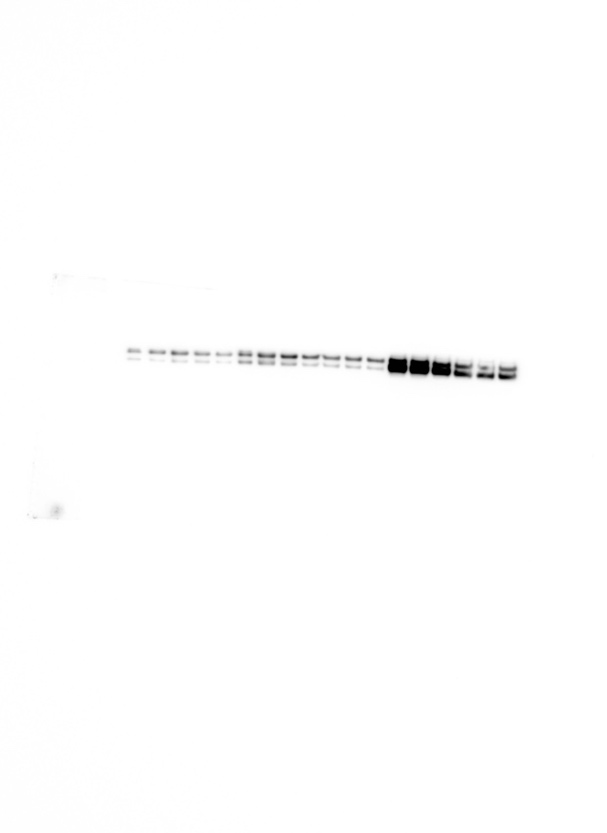


40

50

50

40

pAKT AKT


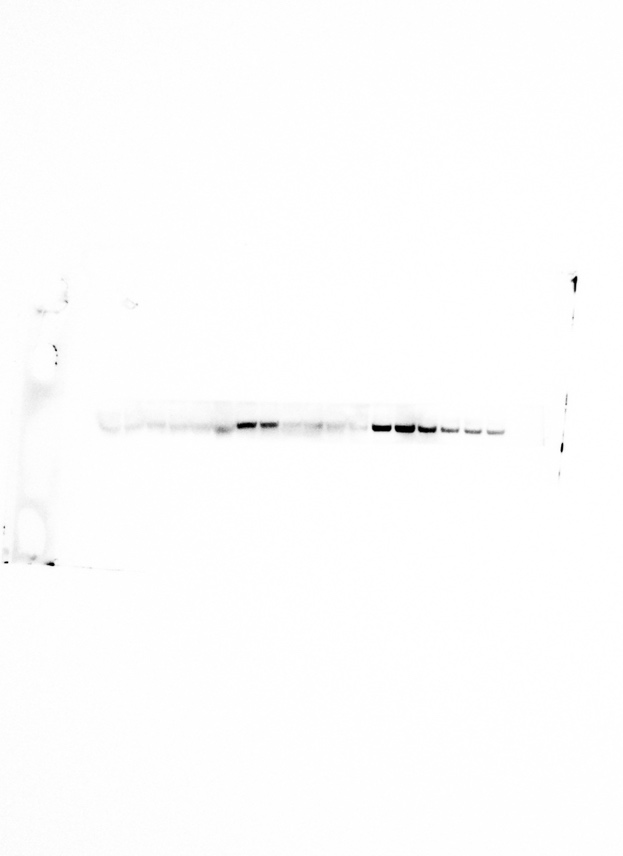

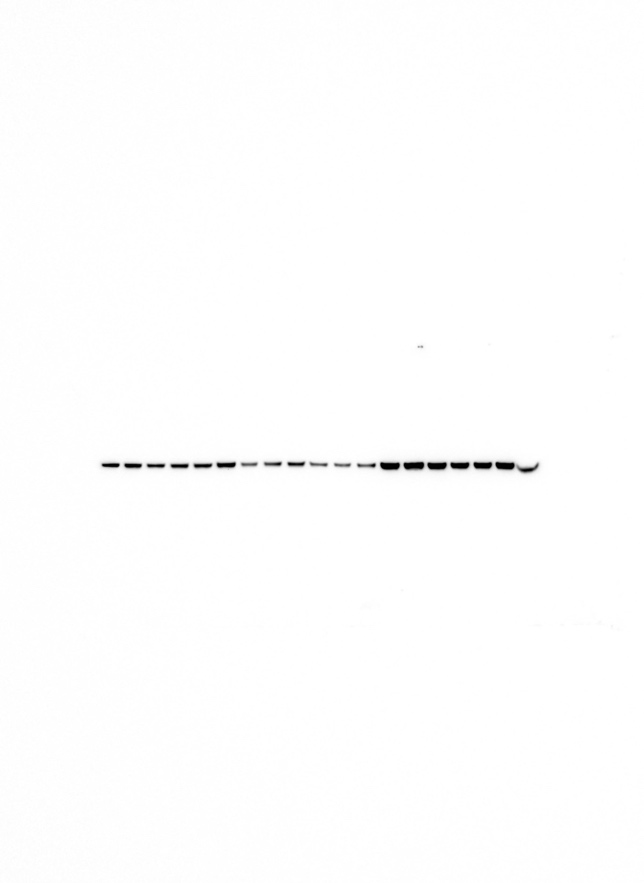


50

60

60

50

SMBO-170

pERK ERK


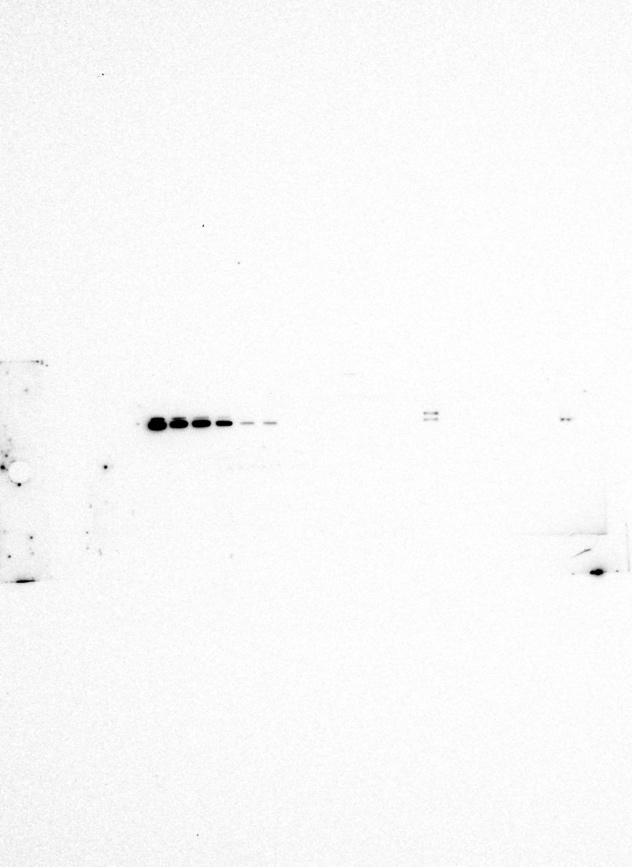

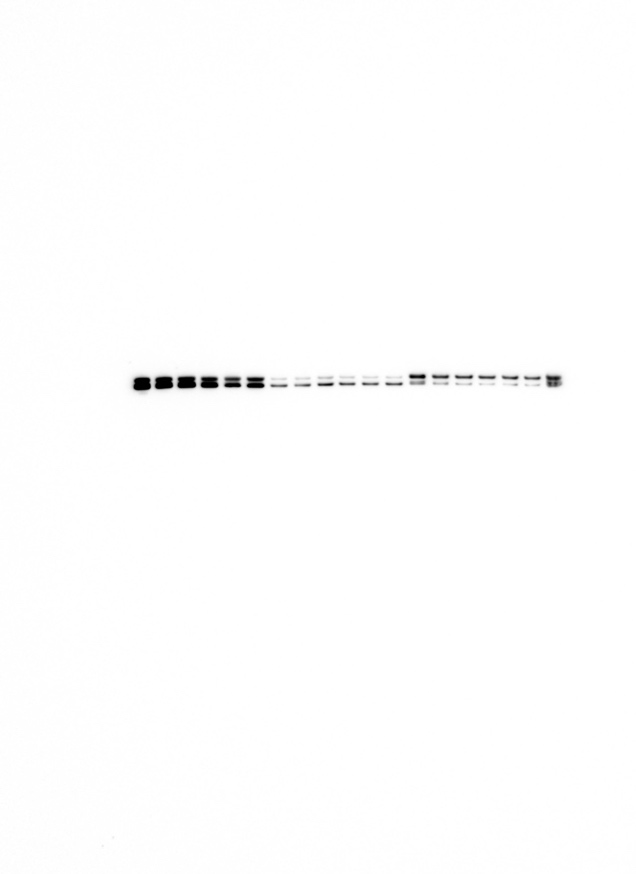


50

40

40

50

pAKT AKT


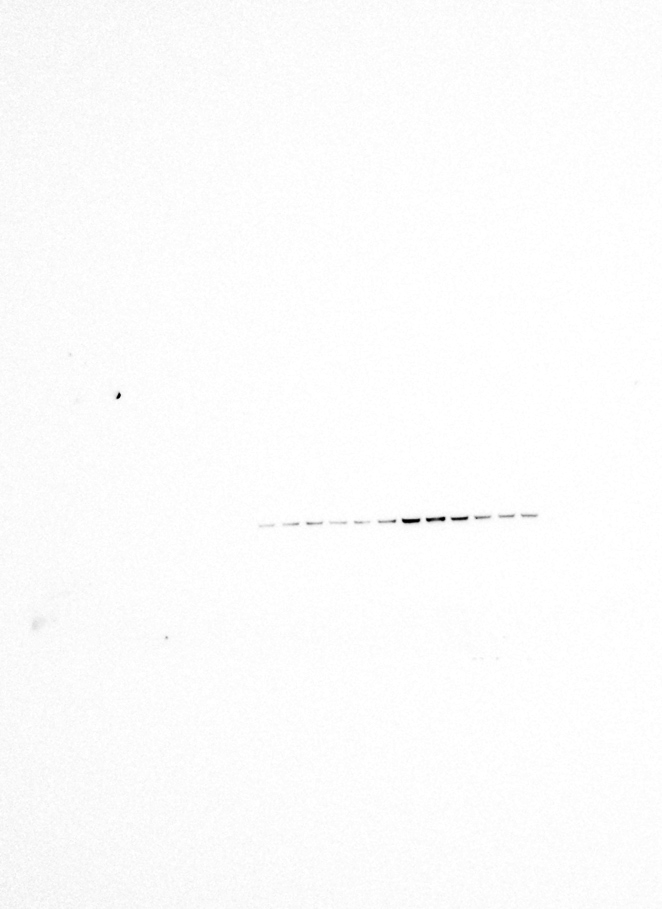

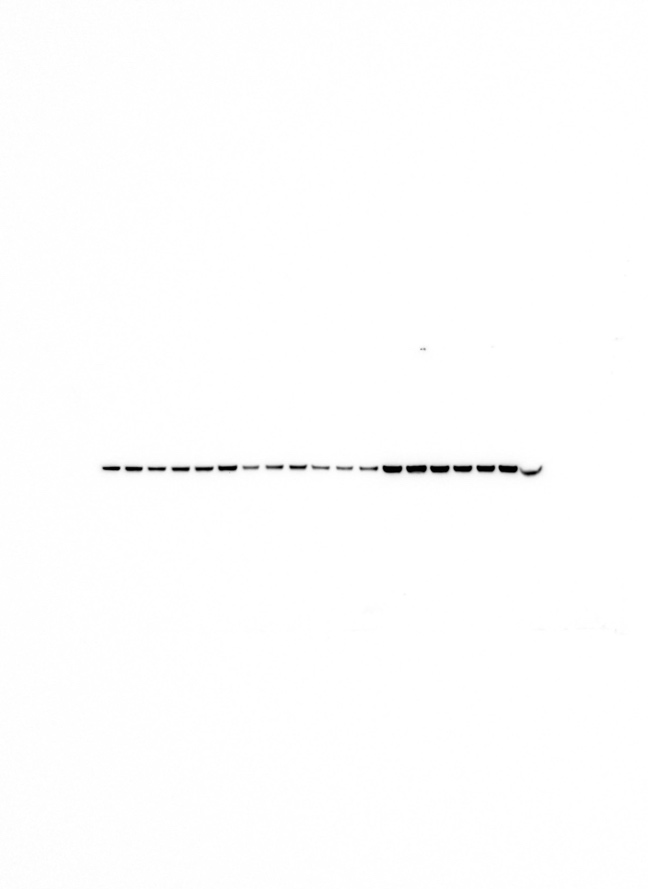


60

50

60

50

SCBO-8

pERK ERK


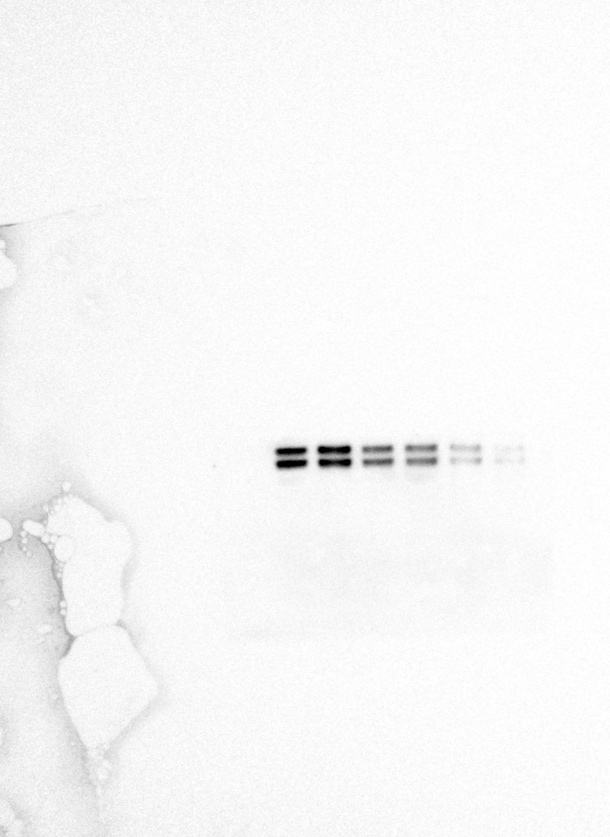

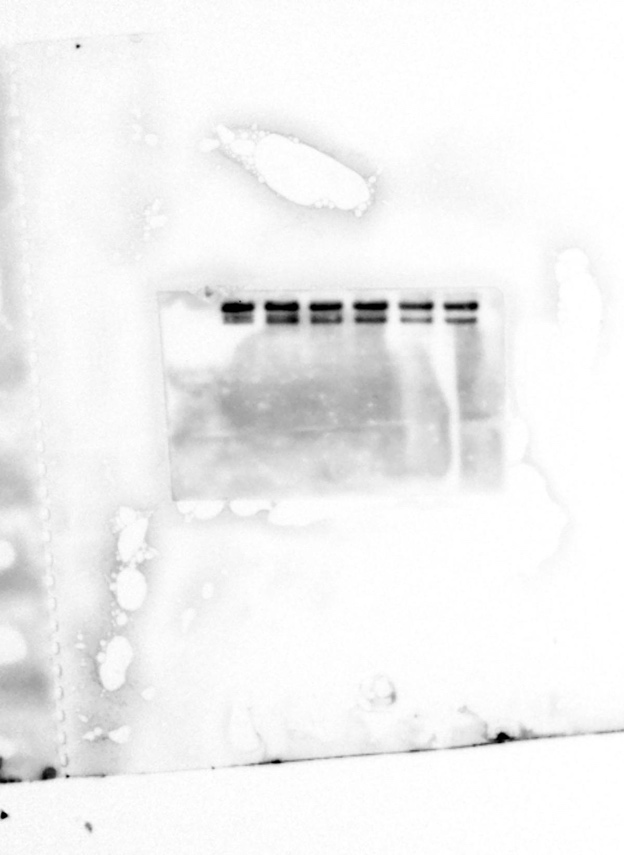


50

40

40

50

pAKT AKT


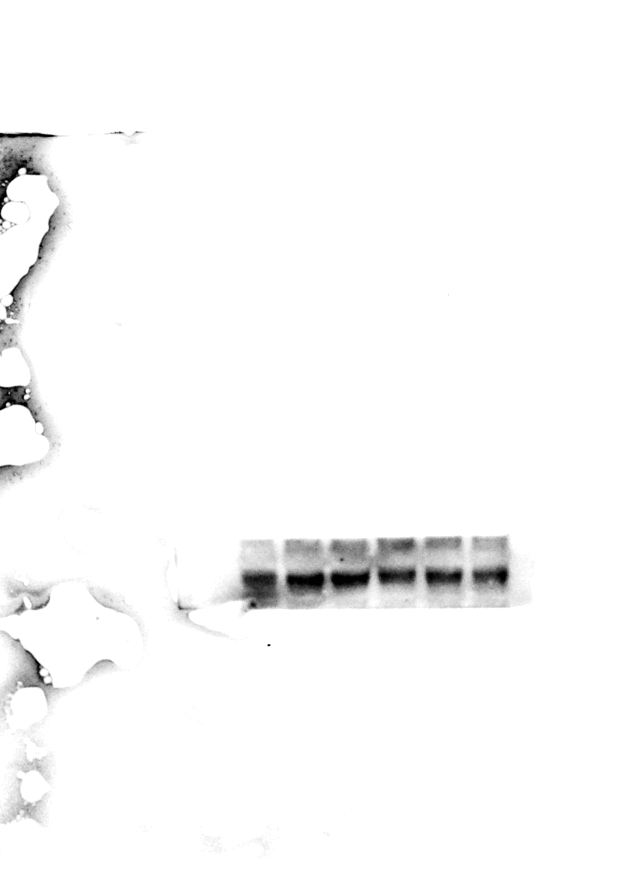

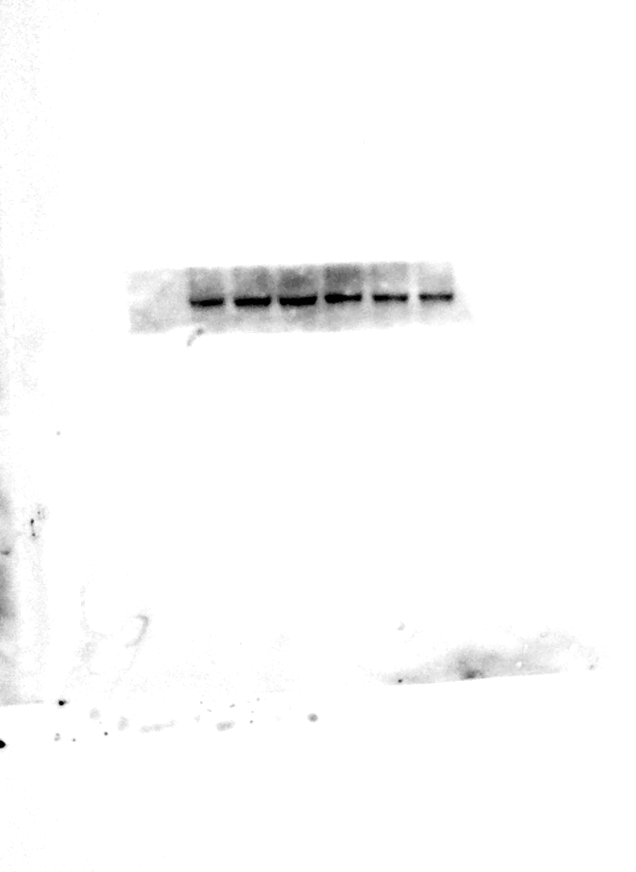


60

50

50

60

SMBO-109

pERK ERK


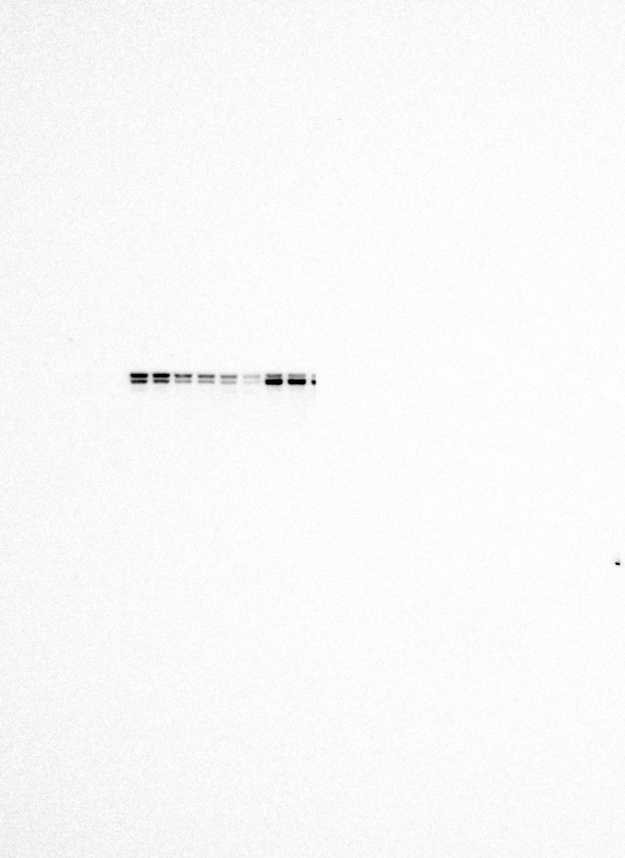

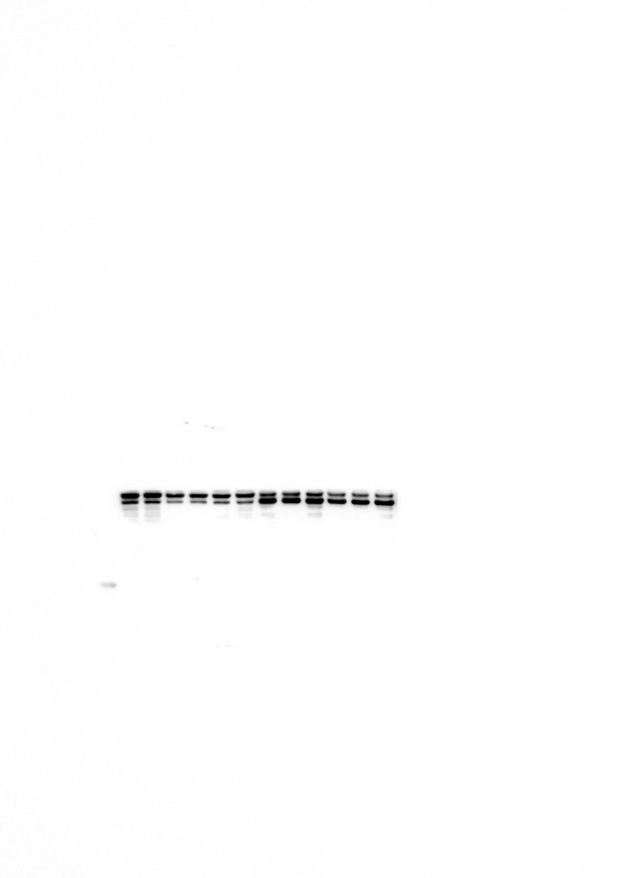


50

40

50

40

pAKT AKT


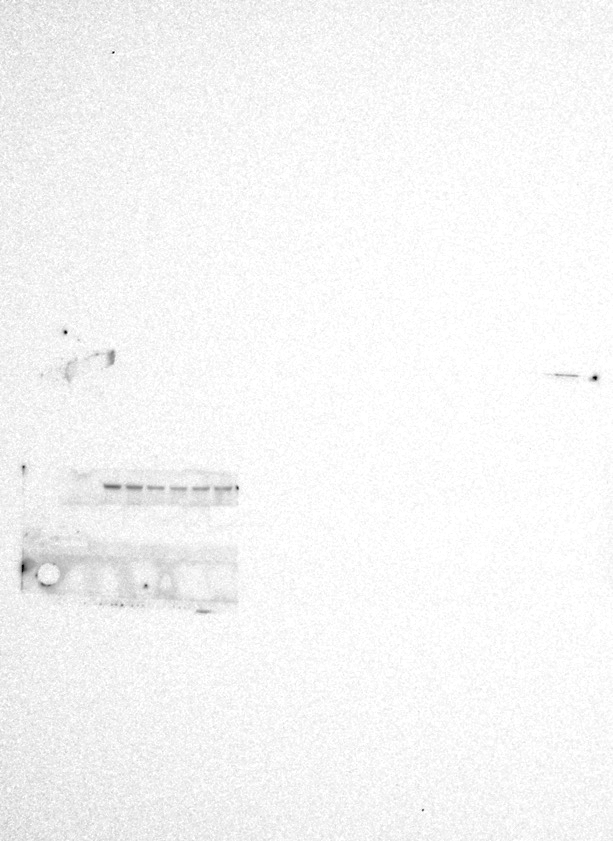

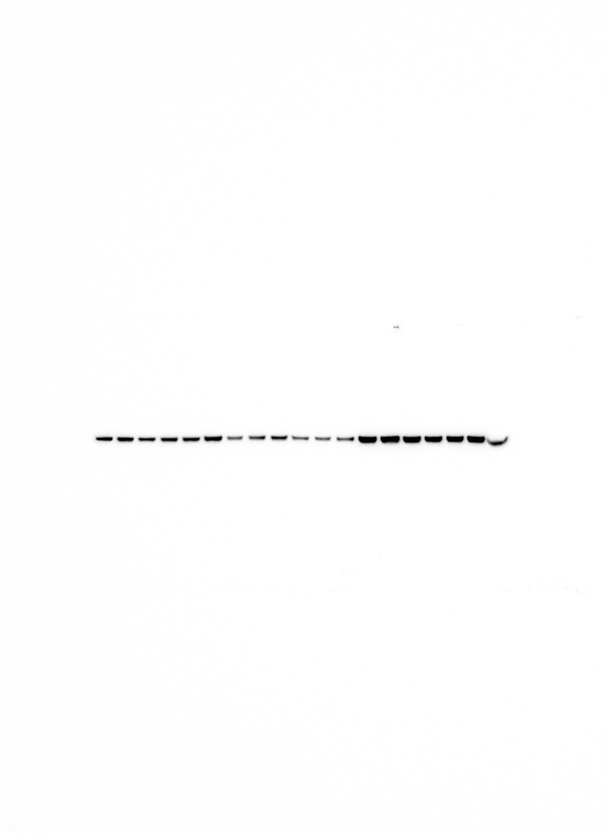


50

60

50

60

SMBO-114

pERK ERK


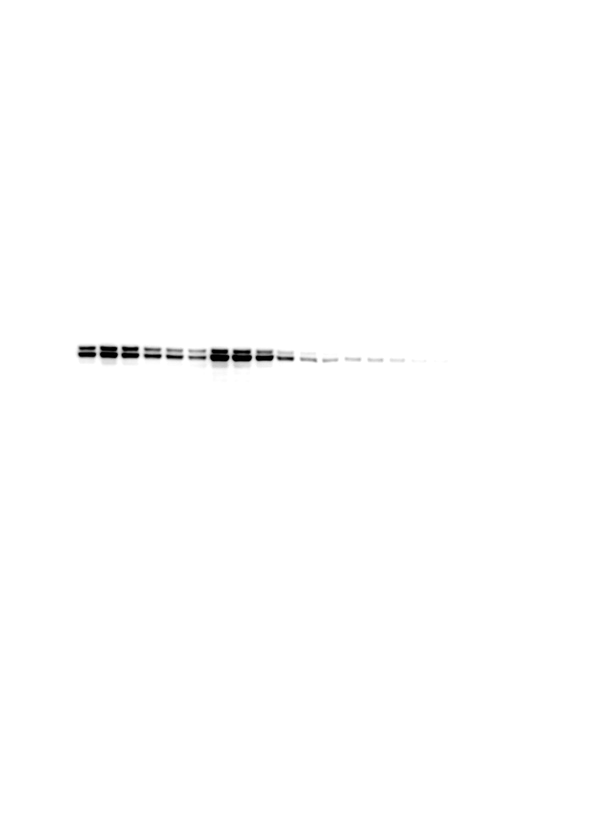

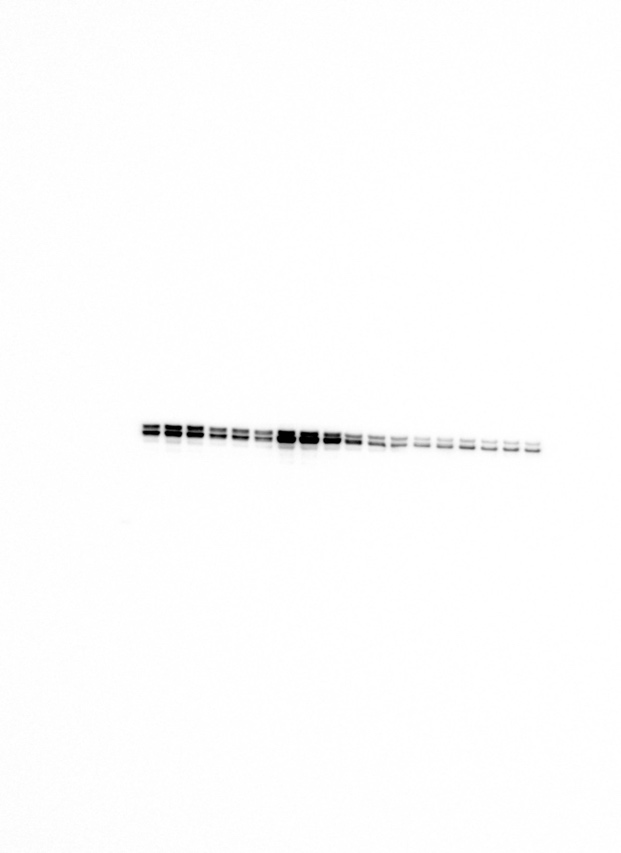


40

50

50

40

pAKT AKT


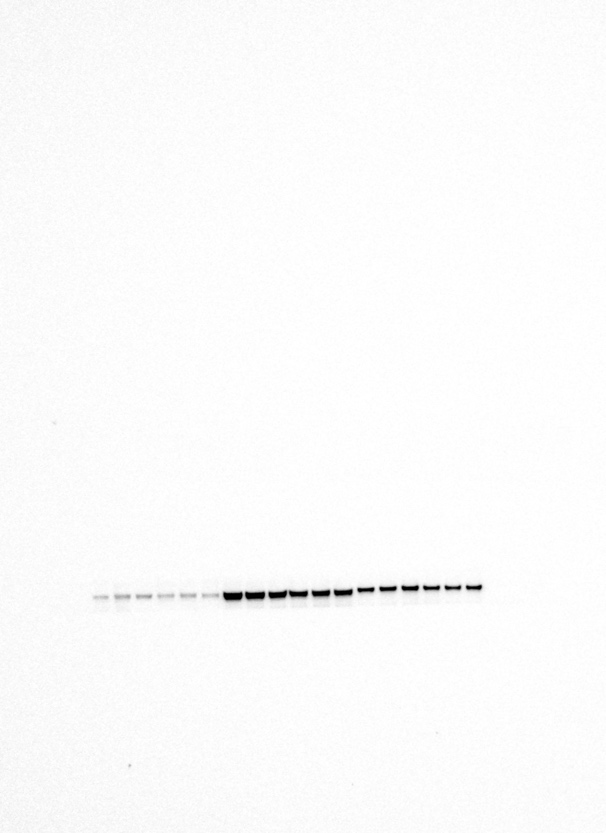

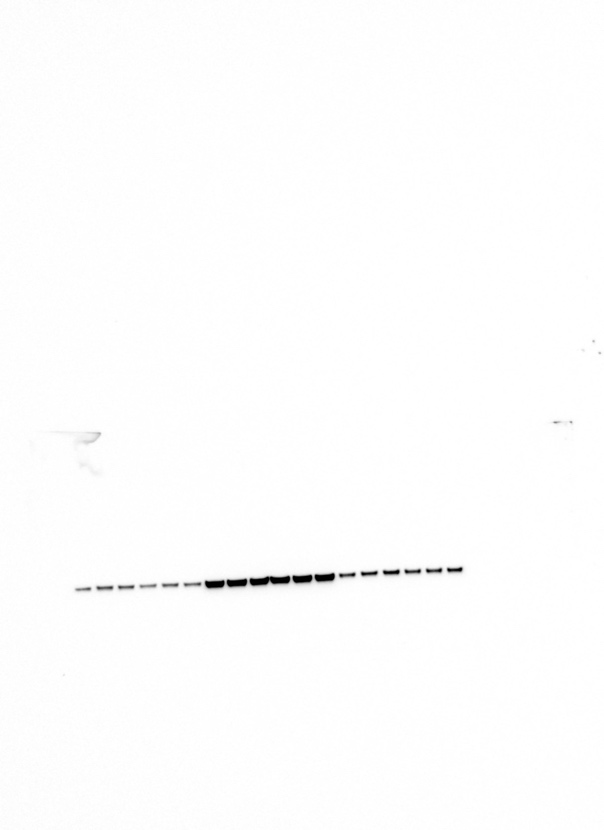


50

60

50

60

SMBO-106

pERK ERK


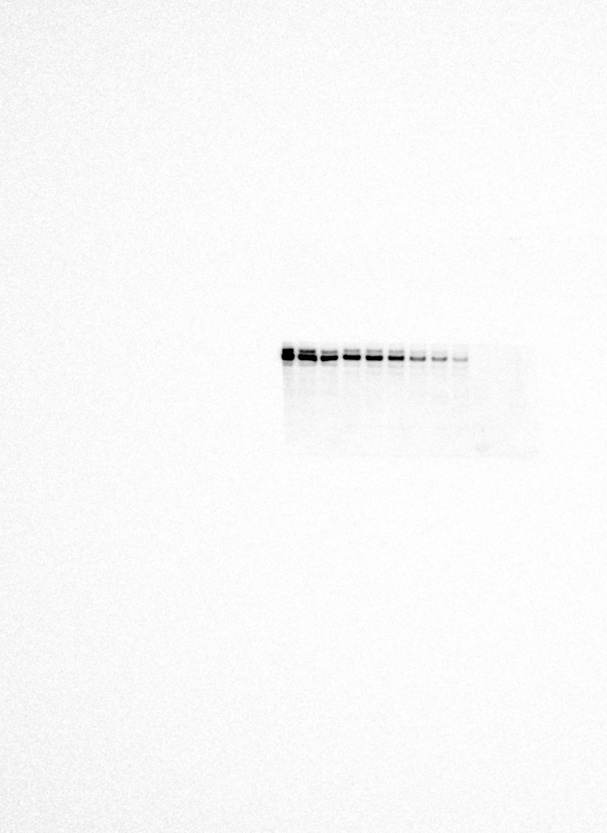

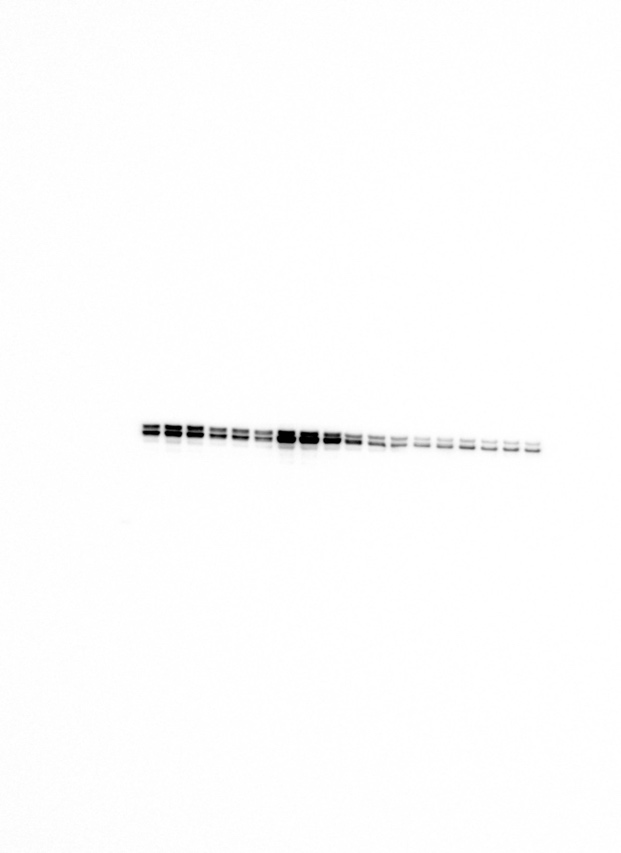


40

50

50

40

pAKT AKT


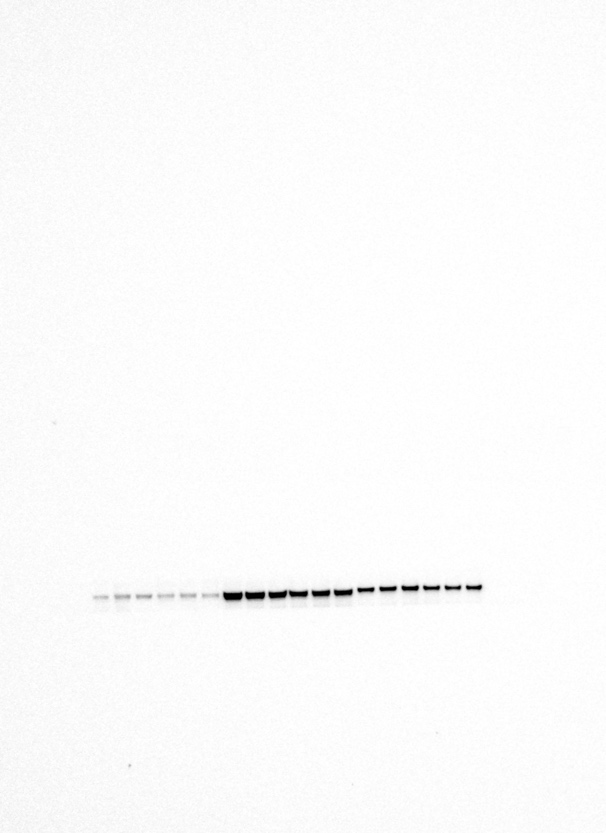

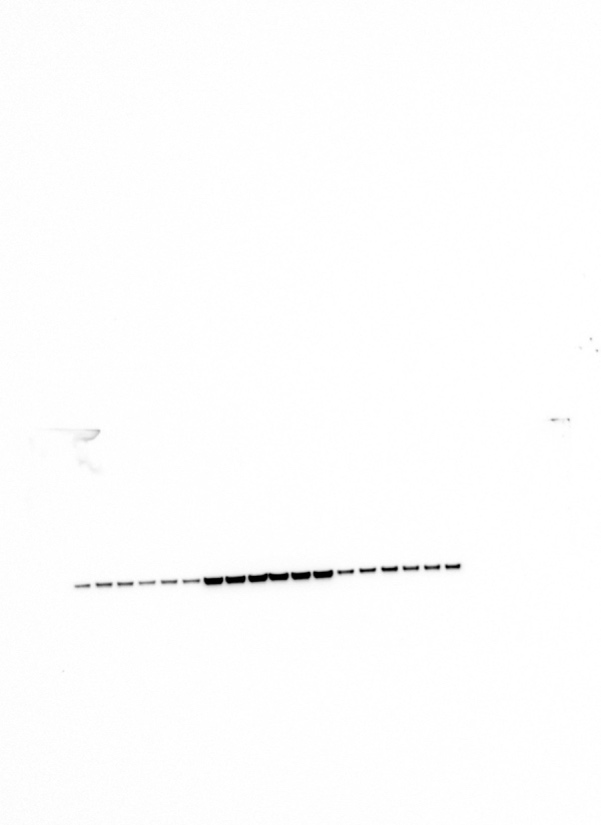


60

50

60

50

**Figure 5D**

HER2 GAPDH

**
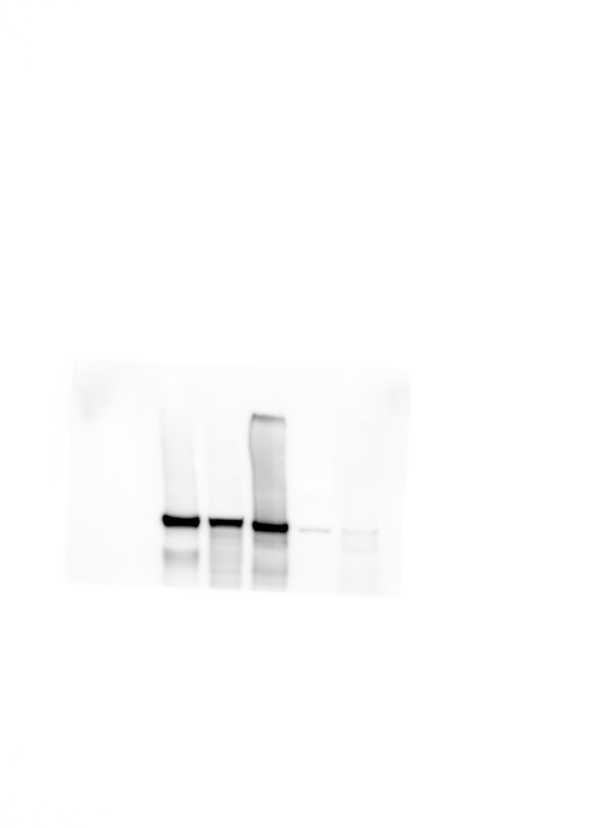

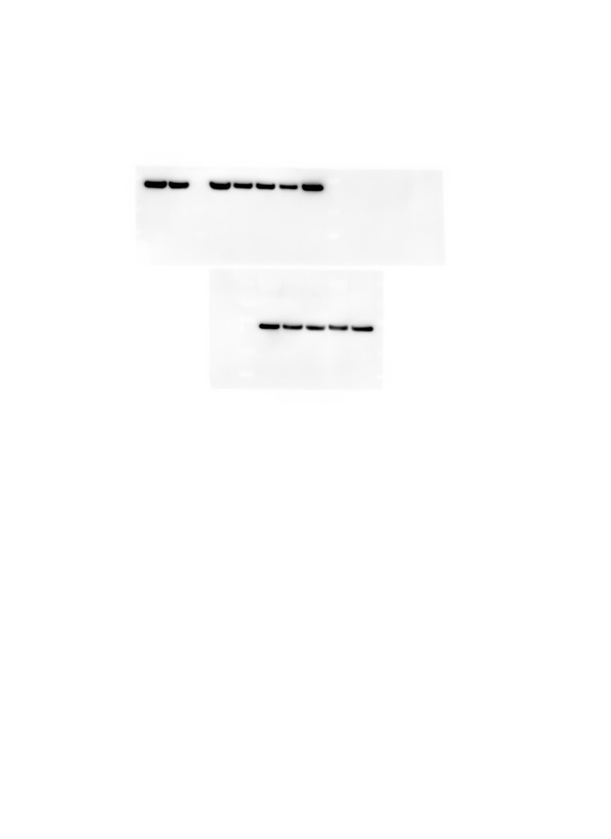
**

30

40

160

110

260

**Figure S7A**

SMBO-106 and SMBO-170 3D figures are the same as those presented in Figure 5A.

SMBO-106 2D

pERK ERK


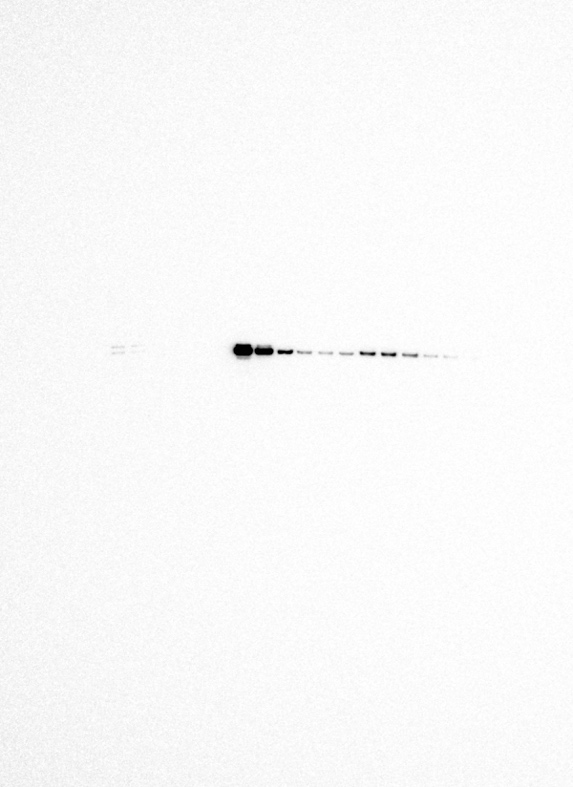

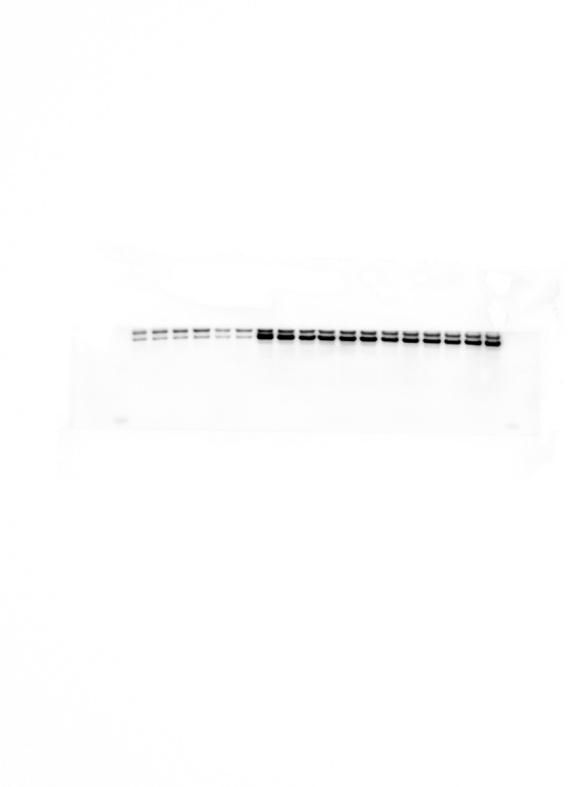


50

40

40

50

pAKT AKT


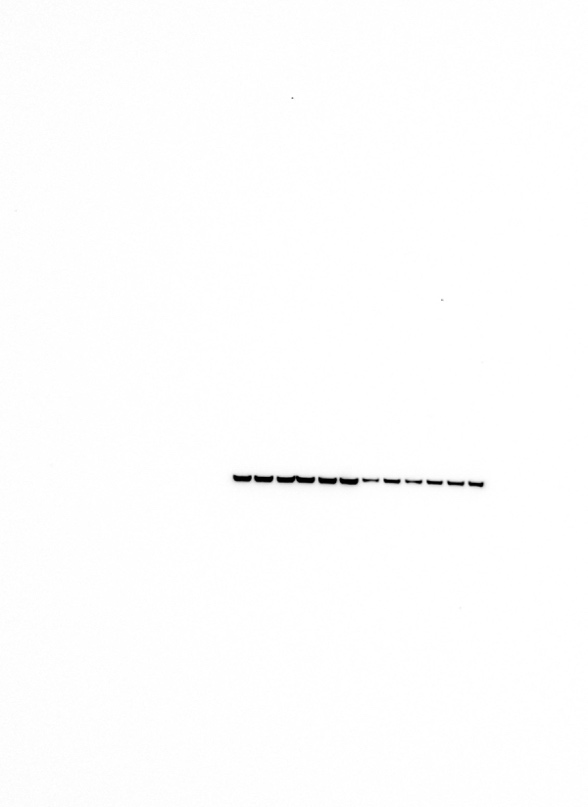

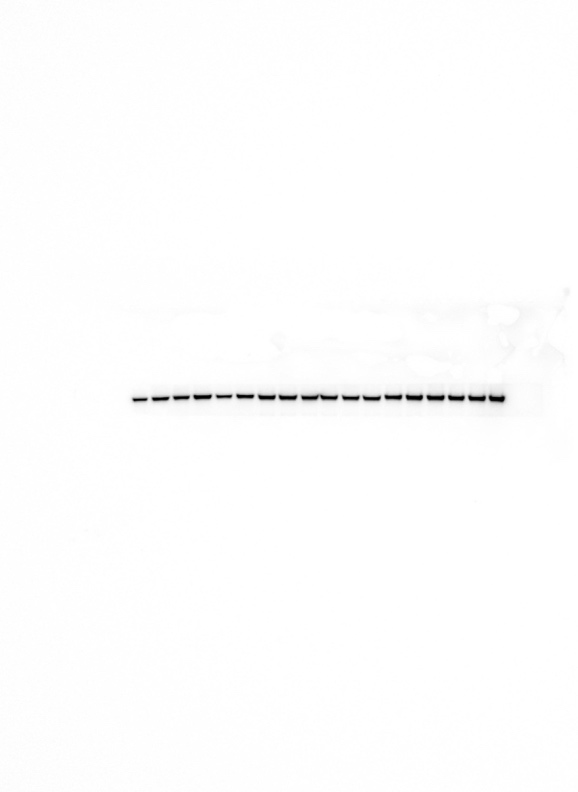


60

50

50

60

SMBO-170 2D

pERK ERK


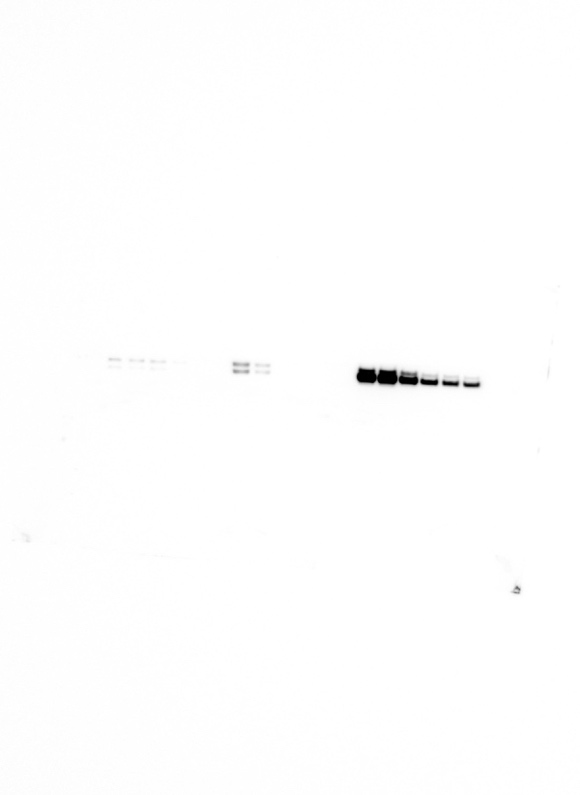

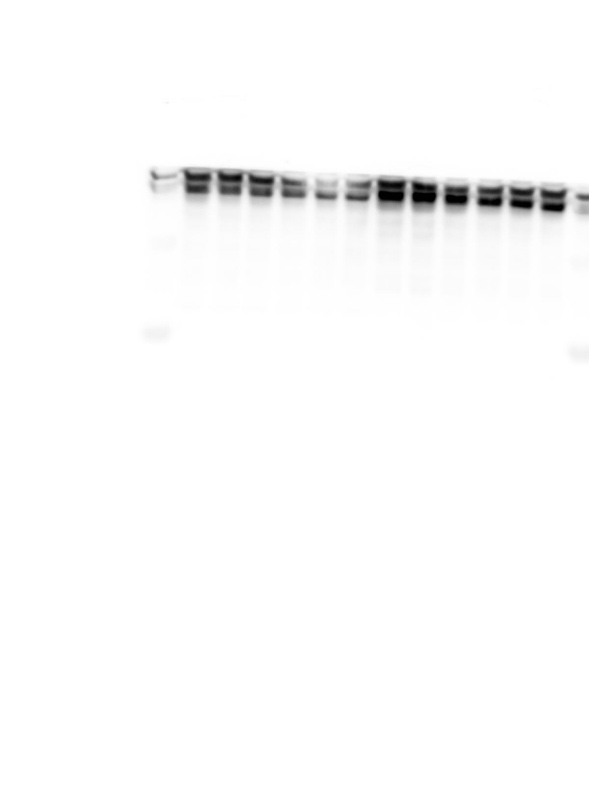


50

40

40

50

pAKT AKT


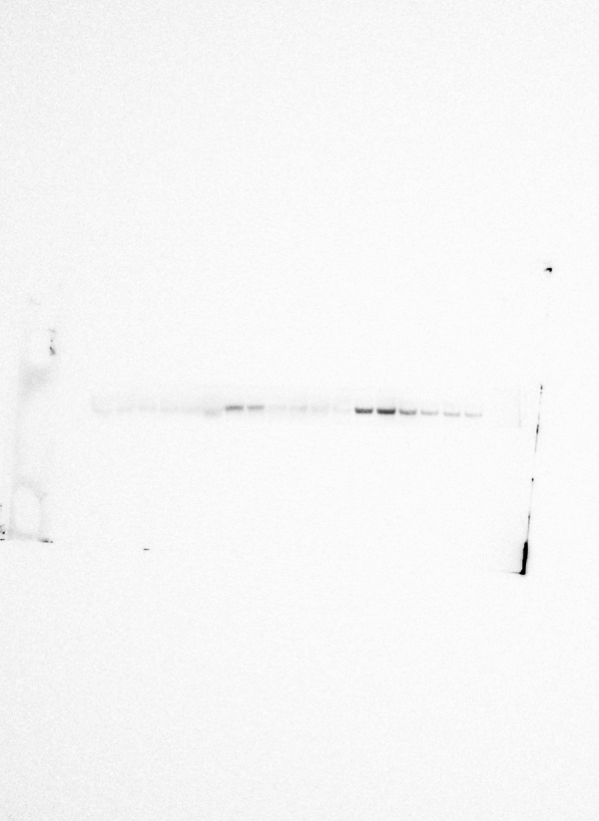

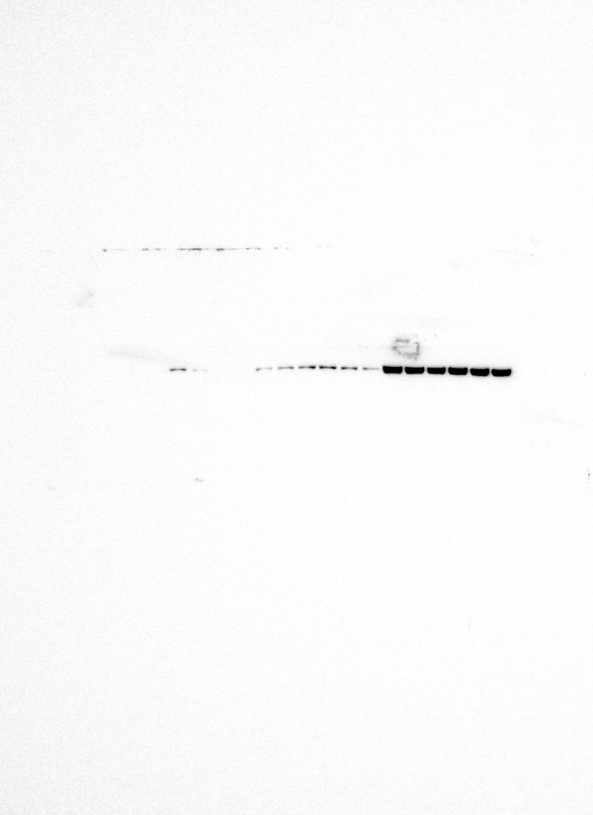


60

50

60

50
